# *Hedychium gardnerianum* Sheph. ex Ker Gawl. from its discovery to its invasive status: a review

**DOI:** 10.1186/s40529-021-00318-5

**Published:** 2021-07-22

**Authors:** Maria João Pereira, Telmo Eleutério, Maria Gabriela Meirelles, Helena Cristina Vasconcelos

**Affiliations:** 1 Biotechnology Centre of Azores, University of Azores, Ponta Delgada, Portugal; 2 Biology Department, University of Azores, Ponta Delgada, Portugal; 3 Department of Physics, Chemistry and Engineering Sciences, University of Azores, Ponta Delgada, Portugal; 4 Centre for Physics and Technological Research, University of Azores, Ponta Delgada, Portugal

**Keywords:** *Hedychium gardnerianum*, Nomenclature, Types, History of introduction, Distribution, Invasion severity

## Abstract

*Hedychium gardnerianum* Sheph. ex Ker Gawl. is one of the 100 world's worst invasive alien species and the research target in areas as diverse as biological control, natural fibres uses, taxonomy or the biological activity of its compounds. This review aimed to clarify the taxonomic status and the native range of *H. gardnerianum* and bring accuracy to the history of its introduction and escape from cultivation through the analysis of the increasing number of accessible digitalized dry specimens and grey literature. The analysis of the available information allowed to conclude that: (a) *Hedychium gardnerianum* is a validly published name, the authority of the name is Sheph. ex Ker Gawl., the species holotype is the illustration published along with the species name, and the Natural History Museum BM000574691 specimen collected in 1815 is the first dried specimen of *H. gardnerianum*; (b) This species is native to the Central and Eastern Nepal, Bhutan, Northeast India and North Myanmar; (c) The species was cultivated at Cambridge Botanical Garden since 1818 and the first known herbarium specimen collected in Europe dates back to 1821; (d) Kathmandu (Nepal) and Khasi Hills (India) specimens are considered two varieties of the same species and the BM000574691 specimen is the lectotype of *H. gardnerianum* var. *speciosum*; (e) Specimens, references, and/or pictures support that *H. gardnerianum* escaped from cultivation at Galicia (Spain), Azores archipelago, Madeira, Tenerife, Cuba, Jamaica, Martinique, Trinidad, Ascension, Mexico, Honduras, Brazil, South Africa, Swaziland, Zimbabwe, Réunion, Mauritius, Australia, New Zealand, Fiji, Hawaii, and Vietnam; and (f) *H. gardnerianum* is a serious pest in Azores, Madeira, Jamaica, Réunion, New Zealand and Hawaii and continues to expand its distribution area in South and Central America, Australia and Southern Africa. This review presents linear raw information compiled with precision, allowing the world databases updating their data but also gives the most detailed information possible to each country/region identifying new regions of concern and updating the invasiveness status in each region.

## Introduction

*Hedychium gardnerianum* Sheph. ex Ker Gawl. (IPNI 2021) is a perennial herb with large branching surface rhizomes producing stems 1–2 m tall; the bright green, long ovate-elliptic (25–45 cm × 10–15 cm) and subsessile leaves are alternately arranged with sheaths clasping the stems; the plant produces terminal cylindrical spikes (25–40 cm long) above the foliage, holding scented bright yellow flowers with a single large bright red stamen, and later orange fleshy capsules with small shiny red seeds included in a crimson aril (CABI 2021a) (Fig. 2). This ornamental species is one of the ‘100 of the World's Worst Invasive Alien Species’ (GISD 2021) with high environmental and economic costs for several countries; nevertheless, the plants and seeds are still marketed worldwide without any advertence or recommendations about the conditions that potentiate its escape from cultivation.

At the end of the twentieth century research on this plant was focused on its physical, chemical, and biological control (CABI 2021a; GISD 2021); in the twenty-first century the research is focused on the effectiveness of in-field *H. gardnerianum* control actions (e.g. Chauchard and Lavergne 2009; Minden et al. 2010a), remote sensing technologies for mapping this invasive species (Asner and Vitousek 2005), modelling its potential distribution (e.g. Baret et al. 2006; Gallardo et al. 2015); investigating the biological activity of its compounds (Medeiros et al. 2003; Rosa et al. 2010; Arruda et al. 2012; Tavares et al. 2020), and its use in cattle feeding (Nunes et al. 2014) or biomaterials production (Eleutério et al. 2017, 2018, 2020).

Current worldwide research on invasive species make use of important biological databases as CABI (2021a), POWO (2021), GISD (2021) or PIER (2021) and great economical and human efforts are put nowadays in the construction of those databases which need to be constantly updated and revised to become sources of reference and avoid lapsus spread in literature. Also, in the last decades we gain access to an increasing number of digitalized documents on databases as Biodiversity Heritage Library (2021), the Internet Archive (2021) or digitalized specimens (e.g. Natural History Museum 2021; AVH 2021). In fact internet has profoundly changed how we produce, use and collect research and information with grey literature (data that is either unpublished or has been published in non-commercial form) playing an increasingly important role (Laurence et al. 2015; UNE 2021). Although finding, accessing and evaluating this material can be a difficult and time-consuming task, the importance of grey literature on research has been recognized (Haddawaya and Baylissb 2015).

The analysis of the currently available information about this important invasive species unveiled some inconsistencies regarding the authority of the scientific name and its synonymy, its native range and the regions where the species escaped from cultivation. Therefore, this review aims to clarify the taxonomic status and the native range of *H. gardnerianum* and bring accuracy to the history of its introduction and escape from cultivation. This review presents linear raw information compiled with precision allowing the world databases to update their data and gives the most detailed information possible to each country/region emphasizing the lack of knowledge to fulfil, identifying new regions of concern and updating the invasiveness status in each region.

## The scientific discovery of *Hedychium gardnerianum*

### Wallich's *Hedychium speciosum* from the Khasi Hills (India)

**Fig. 1:**
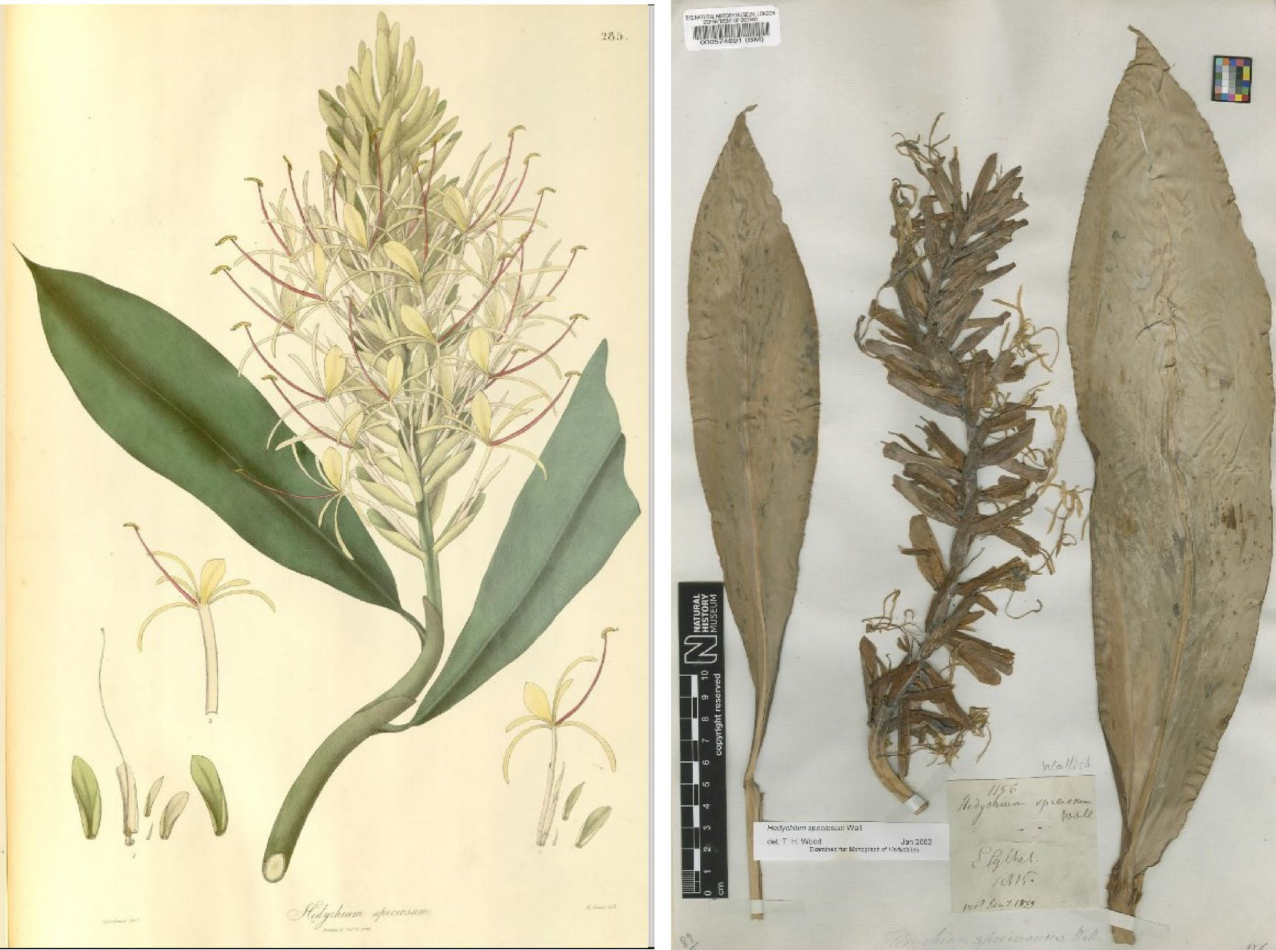
At left, original drawing taken from the first collected specimen sent to Nathaniel Wallich from the Kasia range by Mr. Matthew Robert Smith and printed as Tab 285 in Plantae Asiaticae Rariores (1832). At right, the BM000574691 specimen collected in 1815, presumably by Mr. Matthew Robert Smith (Courtesy of UK Natural History Museum)

In 1820, Nathaniel Wallich, the Calcutta Botanical Garden Director, publishes in *Flora Indica* the description of two new species of *Hedychium*: *H. villosum* and *H. speciosum;* however, no drawing or specimen number is indicated for each species (Carey 1820). When referring to *H. villosum* he states: ‘A native of the mountains North-East of Bengal, from whence our indefatigable collector of plants, Mr Matthew Robert Smith, sent specimens to me in 1815’ (Carey, 1820, p 12). In the next description regarding *H. speciosum* he states*:* ‘A native of the same country [mountains of North-East of Bengal] with the preceding [*H. villosum*], and like all the species flowering in the rainy season’ (Carey, 1820, p 13). Although it is not explicit to *H. speciosum*, we assume that this first specimen was also collected and sent by Matthew Robert Smith in 1815; in fact, at the UK Natural History Museum (2021) botanical collections, the BM000574691 herbarium specimen is identified as *H. speciosum* (Fig. 1) and the BM000574717 herbarium specimen is identified as *H. villosum,* both collected in 1815.

Sanoj et al. (2013) while studying *H. villosum* specimens, also linked the sheet BM000574717 (labelled in Wallich’s hand with the year 1815 but no collector name) with the specimen collected by Matthew Robert Smith and referred by Wallich in 1820 (Carey 1820).

Only In 1832, in *Plantae Asiaticae rariores* book, Wallich publishes a plant draw in Tab 285 to support the plant description published in 1820 (Fig. 1). Later in the 1853 Hooker's journal of botany and Kew Garden miscellany, Wallich links the 1820 description and the 1832 illustration to the ‘first specimen sent by post, from the Kasia range by Mr M. E. Smith, nearly 40 years ago’ (Wallich, 1853, p 370). Again, he does not indicate any specimen number and again we assume that he refers to the BM000574691 specimen collected in 1815. Also, in the 1853 publication, Wallich recognizes that *H. speciosum* and *H. gardnerianum* are the same species and he retains the *H. gardnerianum* name in honour of his friend Edward Gardner.

### Mr Gardner's garland flower from Kathmandu (Nepal)

**Fig. 2:**
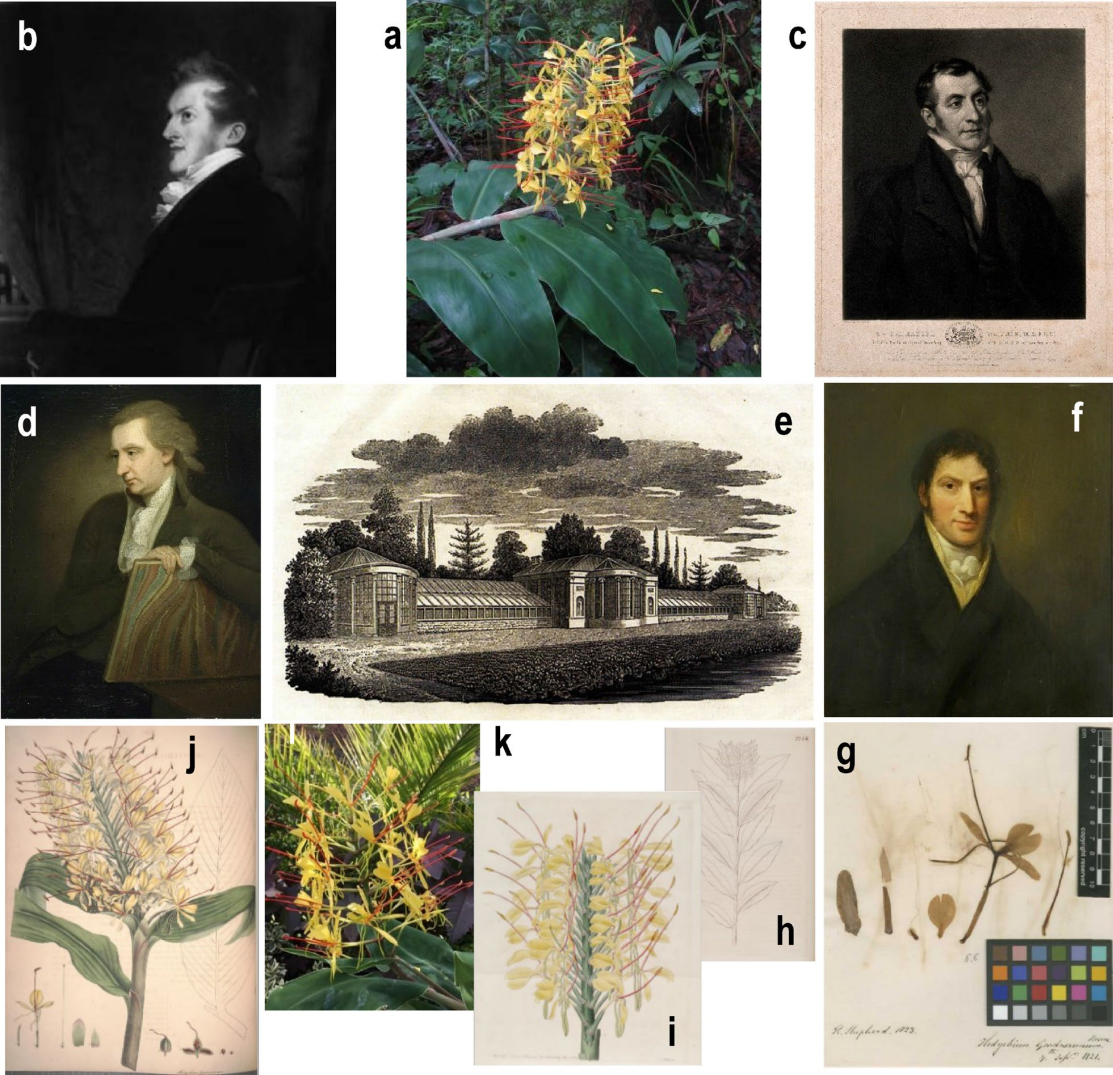
**a** Flowering plant of *Hedychium gardnerianum* in its native habitat at Nepal (Lalitpur district of Kathmandu Valley) (Photographer B. Adhikari) (Flora of Nepal 2021). **b** Edward Gardner (Resident to the Court of Nepal from 1816 to 1829) (Bilder aus Nepal 2021; Fraser-Jenkins 2006); **c** Nathaniel Wallich in 1833 (Director of the Calcutta Botanical Garden from 1817 to 1846) (Welcome Collection 2021; Das Gupta 2011); **d** William Roscoe (Co-founder of the Liverpool Botanic Garden in 1802) (Walker Art Gallery 2021a; Roscoe 1833); **e** Conservatory in 1808 at Liverpool Botanical Garden in Mount Pleasant (1802–1831) (Kaye 1820; Law 2007). **f** John Shepherd (curator of Liverpool Botanic Garden at Mount Pleasant from 1802 to 1831) (Greenwood et al. 2018; Walker Art Gallery 2021b); **g** First dried specimen made by Henry Shepherd in the second year of plant blooming (LINN-HS 8.8—The Linnean Society of London 2021); **h**, **i** Illustrations published in February, 1824 by Ker-Gawler along with John Shepherd first description of *H. gardnerianum*; **j** Illustration published in 1828 by William Roscoe, on his book about the Monandrian plants of the order Scitamineae. K. *H. gardnerianum* at Walled Garden in Croxteth Hall and Country Park in the summer of 2017 (Cable 2017)

During the latter end of 1817 and the whole 1818, Edward Gardner (the first Resident to the Court of Nepal from 1816 to 1829) and his team, will have collected an *Hedychium* plant at the Kathmandu Valley and sent it to Wallich in India (Ker-Gawler 1824; Roscoe 1828; Smith 1832; Fraser-Jenkins 2006). In 1819, Wallich sends a living plant of Mr Gardner’s Garland-flower to William Roscoe at the Liverpool Botanical Garden under the name *H. gardnerianum*. The plant arrived in September 1819 and it grew at the conservatory under the care of the Garden Curator John Shepherd, blooming on 4th October 1820 (Roscoe 1828; Law 2007; Greenwood et al. 2018). The produced seeds germinated and certainly due to the scarcity of plants produced in the first year, the nephew of the Curator and also sub-curator Henry Shepherd, takes only a flower on 7th September 1821 (allowing the other flowers developing seeds) to produce much probably the first herbarium specimen (LINN-HS 8.8.) of this species collected in the UK (Roscoe 1828; Greenwood et al. 2018; The Linnean Society of London 2021). In 1824, Ker Gawler uses the manuscript notes on *H. gardnerianum* made by John Shepherd and publishes this first description along with the scientific name ‘*Hedychium gardnerianum’* in the horticultural magazine 'The Botanical Register', accompanied by an illustration of the flowering plant made from another specimen grown in Mr Hatfield greenhouse at the Alpha Cottages (Ker-Gawler 1824; Law 2007) (Table 1). In 1828, Roscoe also published an illustration of this species in bloom on his book about the Monandrian plants of the order *Scitaminea.* Fig. 2.

**Table 1:** *Hedychium gardnerianum*: authority variations, incorrect spelling of authority and absence of authority in digital databases of reference

Authority	World digital databases	Nations/Countries’ digital databases
Sheph. ex Ker Gawl	IPNI (2021)	
Ker Gawl		Swaziland's Alien Plants Database (2021)
Ker Gawl		Flora of Zimbabwe (Hyde et al. 2021) Flora of New Zealand (2021)
Sheppard ex Ker Gawl	The Plant List (2013) Plants of the World Online (2021) CABI (2021a) Encyclopedia of Life (2021) Catalogue of Life (2021) Reflora (2021)	India Biodiversity Portal (2021) Borbonica – Réunion (2021) Atlas of Living Australia (2021) Flora-on Açores (Portugal) (2021) Alien Invasive Plants List for South Africa (2021)
Shepard ex Ker Gawl	Integrated Taxonomic Information System (2021)	USDA Plants DataBase (2021)
Sheppard ez Ker Gawl		Nepal National Herbarium and Plant Laboratories KATH (2020)
–	Barcode of Life Data System v^4^ (2021) Global Invasive Species Database (2021) Royal Horticultural Society (U.K.) (2021)	

In the protologue (Bot. Reg. 9: t. 774 1824) the author is cited as Sheppard but refers to John Shepherd, curator of Liverpool Botanical Garden (IPNI 2021)

### *Hedychium speciosum* or *Hedychium gardnerianum*?

We can argue that the name *H. speciosum* was not validly published in 1820 since no illustration or dried specimen (nomenclatural type specimen) is clearly identified: 'My examination of this stately plant has hitherto been confined to a well-preserved spike and a few leaves only, which however point it out as the largest of the genus’ (Carey 1820, pp 13–14).

Only in 1832 Wallich would validly published *H. speciosum* adding an illustration to the previous description, nevertheless, for the same species, the name *H. gardnerianum* was already validly published in 1824 by Ker-Gawler. In addition Wallich recognises in 1853 that they are the one and the same species and accepts the name *H. gardnerianum.*

In a second point of view, using the information published later by Wallich in 1853 where he clarifies that the illustration published in 1832 was made from a dried specimen that Matthew Robert Smith collected in 1815 and assuming that the 1815 BM000574691 specimen is, in fact, the specimen observed by Wallich, this specimen could be considered the lectotype of *H. speciosum* name and this name would be accepted (Natural History Museum 2021).

**Fig. 3:**
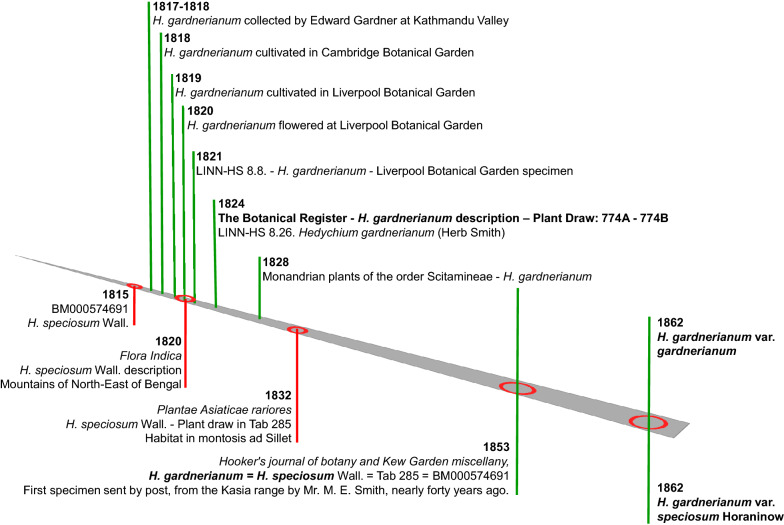
*Hedychium gardnerianum* timeline of nomenclatural events

However, even we accept this reasoning, there are at least two main motives to propose *H. gardnerianum* as *nomina conservanda:* the extended use of *H. gardnerianum* name (Table 2) and the expressed will of Nathaniel Wallich to retain the *H. gardnerianum* name: ‘The magnificent series of specimens, even as to colour, preserved by Drs. Hooker and Thomson, with the fine drawing of the flower and the excellent figure in Roscoe's work, prove that my *H. speciosum* and my *H. gardnerianum* are identically one and the same species. I retain the latter name, being that of a very valued and honoured friend, who, himself ardently attached to flowers and gardening, has done a great deal of service to the cause of botany in its most extended sense’ (Wallich, 1853, p 370) (Fig. 3).

**Table 2:** Presence of *Hedychium gardnerianum* and/or *Hedychium speciosum* names in digital databases of reference (retrieved on June 21, 2021)

Digital source	*Hedychium gardnerianum*	*Hedychium speciosum*
Browser: Google	63 700	853
Nepal National Herbarium and Plant Laboratories-KATH	+	−
India Biodiversity Portal	+	−
Myanmar (Tanaka et al. 2016)	+	
CABI Invasive Species Compendium	+	−
Global Invasive Species Database	+	−
European Vegetation Archive	+	−
Royal Horticultural Society (U.K.)	+	−
Flora-On Açores (Portugal)	+	−
USDA Plants DataBase	+	−
Reflora	+	−
Flora of New Zealand	+	−
Atlas of Living Australia	+	−
Borbonica—Réunion	+	−
Flora of Zimbabwe	+	−
Alien Invasive Plants List for South Africa	+	−
Barcode of Life Data System v4	+	−
Integrated Taxonomic Information System	Accepted	−
Catalogue of Life	+	+
Encyclopedia of Life	58 media/96 data/12 articles	7 data/2 articles
The plant list 2013 / Plants of the World Online	Accepted	Accepted

+ Present; − Absent

### Kathmandu and Khasi Hills specimens as two varieties of the same species

Horaninow (1862) and Sanoj (2011) consider that the specimens collected in these two regions are varieties of the same species; however, although Wood et al. (2000) emphasises that the most important factor in the evolution of *Hedychium* genus is geographic and ecological isolation, the geographic range of these two varieties needs to be established: *H. gardnerianum* var. *gardnerianum* from Kathmandu (Nepal) and *H. gardnerianum* var. *speciosum* (Wall.) Horan. from the Khasi Hills (India) (Fig. 3). At the moment the illustration published in 1824 in The Botanical Register is the holotype of the species *H. gardnerianum* and its variety *gardnerianum,* while the BM000574691 specimen is the lectotype of *H. gardnerianum* var. *speciosum* (Ker-Gawler 1824; Natural History Museum 2021)*.* However, Roscoe in 1828 states that, before September 1819 he received a dried specimen of *H. gardnerianum* under the name *H. excelsum* send by Wallich from Calcutta, and if this specimen did not disappeared in the 1940 bombing raids on Liverpool, it could be chosen to be the lectotype specimen of *H. gardnerianum*. At the moment the extant Wallick herbarium specimens are candidates and the formal work of lectotypification need to be done.

## Native range of the species

*H. gardnerianum* is native to the Central and Eastern Nepal (Shrestha et al. 2018), Bhutan (Noltie 1994), Northeast India (e.g. Nirola and Das 2017) and North Myanmar (Tanaka et al. 2016) (Table 3).

**Table 3:** Native distribution of *Hedychium gardnerianum*

	Regions	Reference and/or Place, Collector (c)	Herbarium specimens	Collection year
Bhutan	Chukka		**E**00507940	1979
	Punakha		**E**00507938	1914
	Trongsa	Tangsibji Hydro Energy Limited (2015)		
Nepal	Bagmati Pradesh	Kathmandu Valley E. Gardner (c)	**LINN-HS** 8.26	[1817—Before Sept. 1819^a^]
		Wallich (1853) Fraser-Jenkins (2006)	**BM**000589480	[1817- 1819]
			**E**00886869	2016
	Gandaki Pradesh	Manaslu Expedition (2008) (c)	**E**00644827	2008
	Province nº 5	Gubhaju and Gaha (2019)		
Northeast India	Arunachal Pradesh	Basak et al. (2014)		
	Assam	Baruah and Choudhury (2014)	**MKB**-499	2014
	Darjeeling	Nirola and Das (2017)		
	Manipur	Daimei and Kumar (2011)	**K**17103.000	1946
	Meghalaya	Wallich (1820) The Khasi Hills Matthew Robert Smith (c)	**BM**000574691	1815
	Meghalaya Nagaland	Wallich (1853) The Khasi Hills W. Gomez and Francis de Silva (c)	**K**001124174 (n. 6550)^b^	[1821–1832]
		Jain and Prakash (1995)	**E**00507942	[1847–1851]
		Bose (2015–2016)		
	Sikkim	Wallich (1853)	**E**00507941	1992
North Myanmar	Kachin	Tanaka et al. (2016)		

Herbaria: LINN-HS (The Linnean Collections-The Smith Herbarium 2021); BM (Natural History Museum 2021); E (Royal Botanic Garden Edinburg 2021); MKB (Department of Life Science and Bioinformatics of Assam University); K (Kew Royal Botanic Gardens 2021). [yyyy-yyyy] Estimated years of collection

^a^Roscoe (1828)

^b^The Wallich Catalogue Online (2021)

*H. gardnerianum* is not native to Vietnam (Tan et al. 2012) or Thailand (Wongsuwan and Picheansoonthon 2011, 2012). According to Wongsuwan and Picheansoonthon (2011) some herbarium specimens previously collected from north-eastern Thailand, were erroneously identified as *H. gardnerianum* instead of *H. neocarneum* T.L.Wu, K.Larsen and Turland. But *H. gardnerianum* was recorded by Tanaka et al. (2016) from north Myanmar corresponding to the eastern limit of the natural distribution of this species.

From 1815 to 1858 the British territory of Bengal included Sylhet (Bangladesh) and the Khasia Hills (India) (The Map Archive 2021) and down to 1868 ‘Khasia’ was under the Judge of Sylhet (Watson 2013). In 2015, the species was not present at Khadimnagar National Park northeast of Sylhet (maximum altitude of 50 m) (Uddin 2015), in fact the alluvial lowlands of Bangladesh are not the habitat of this species. Considering that Wallich distinguished between ‘Sillet,’ and the ‘Mont. Sillet’ or ‘Mont. Sillet vicinae’ by which he indicated Khasia (Watson 2013), the Wallich’s specimens from ‘North-East of Bengal’ (Carey 1820), ‘montosis ad Sillet’ (Wallich 1832) or ‘Mt. Sillet’ (6550 / K001124174—Kew Royal Botanic Gardens, 2021) indicate the Khasi Hills (the bordering hill regions of Meghalaya at India and not the Sylhet region of Bangladesh). ´The Kasia range’ is later mentioned by Wallich in 1853.

Although under the name *H. speciosum,* the species is considered endangered in the Red Data Book of Vascular Plants of Bangladesh (Khan et al. 2001), we have not found any dry specimens, photographs or other information about the presence of this species in the present territory of Bangladesh, and at the moment Bangladesh should not be considered belonging to the natural area *of H. gardnerianum* distribution.

## Travelling as an ornamental plant

**Fig. 4:**
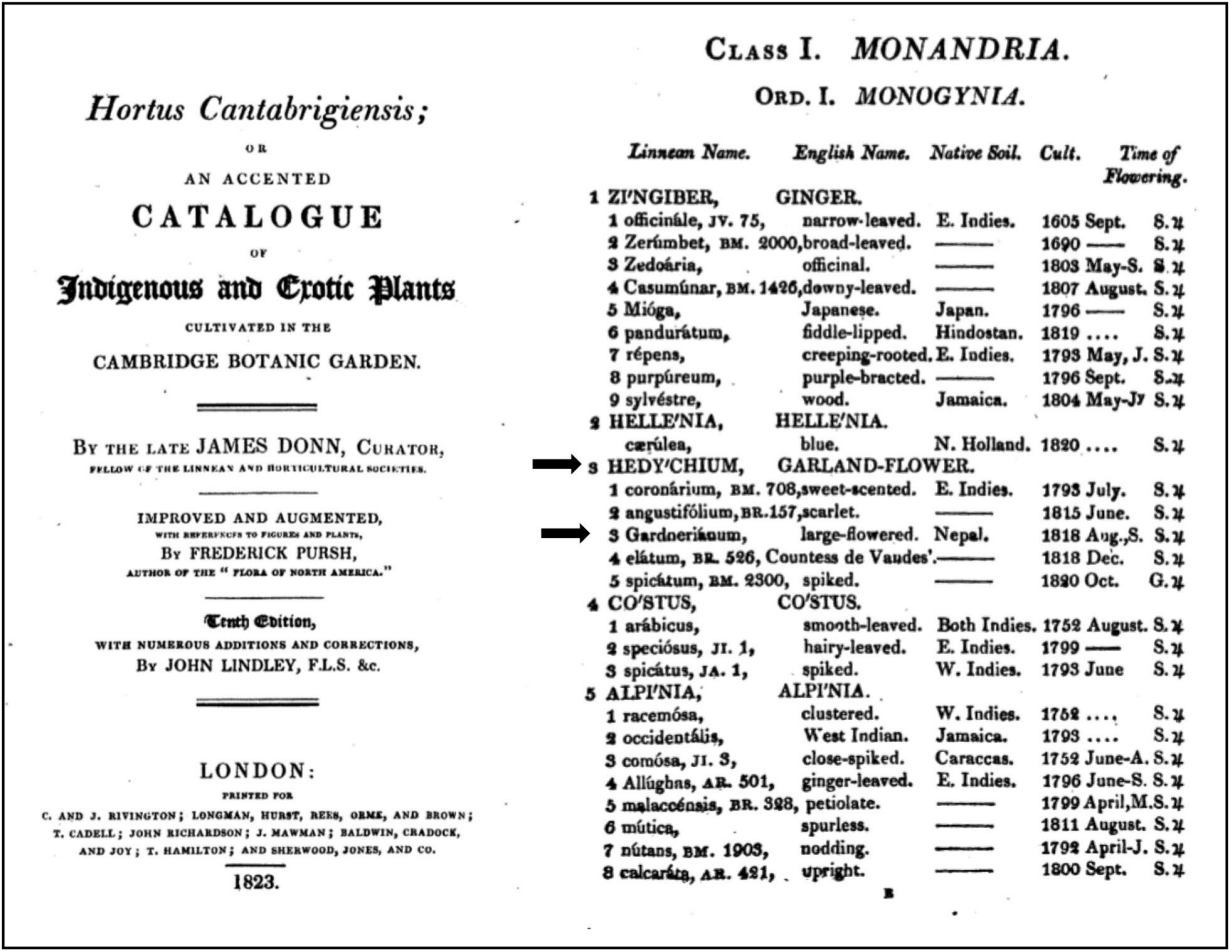
The 1823 catalogue of plants cultivated in the Cambridge Botanical Garden indicate that *H. gardnerianum* is cultivated on that garden since 1818, which change the official arrival date to Europe from 1819 to 1818

Soon after the publication of *H. gardnerianum* name in February 1824, several magazines give notice of this very ornamental plant, e.g.: in England (Tilloch and Taylor 1824), in France (Brongniart 1824), and in Germany (Bernhardi and Völker 1825). From England, the plant is quickly distributed to several European gardens, e.g. Fromont Garden (Bodin 1824) and to their overseas countries or colonies of influence, but according to the Calcutta Royal Botanic Garden Report (Wallich 1840), besides the Liverpool garden and other English gardens (e.g. Royal Botanic Garden of Edinburgh), many seeds and plants were sent to France, North America, Egypt, other parts of India (Chennai, Mumbai, Sri Lanka), China, Malay countries, Australia, islands of Réunion and Mauritius and the Cape (South Africa) and *H. gardnerianum* plants and/or seeds could have been sent to those destinations. In fact, the 1823 catalogue of plants cultivated in the Cambridge Botanical Garden indicates that *H. gardnerianum* is cultivated in that garden since 1818 (Fig. 4). This finding changes the official arrival date to Europe from 1819 to 1818, puts this species as one of the first to be collected by Gardner and sent to Europe (Lindley et al. 1823, 1826; Sweet 1826) and shows the use of this name before its valid publication in 1824.

**Fig. 5:**
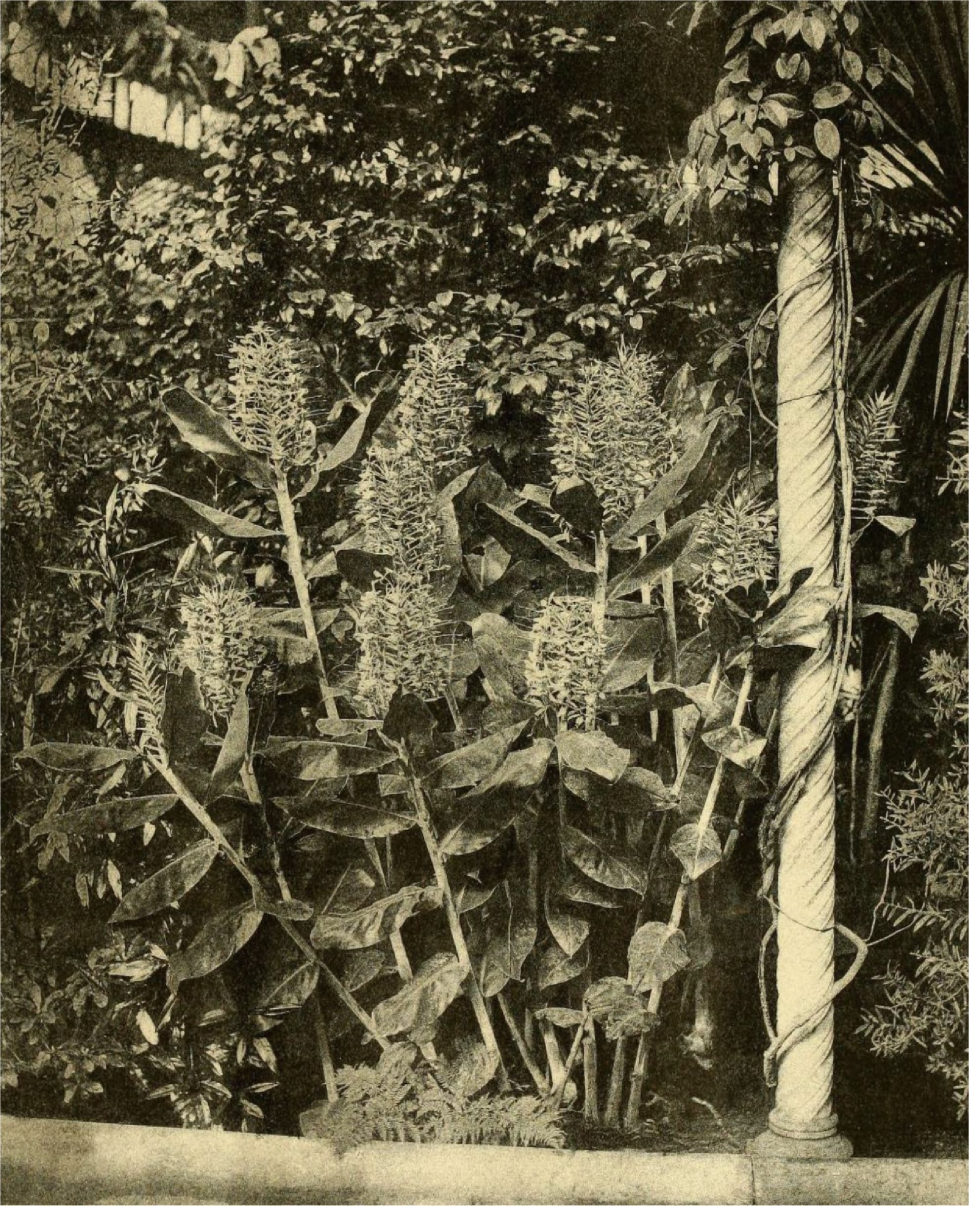
Ink-photo (Sprague & Co.) of *Hedychium gardnerianum* flowering at Glasgow Botanic Garden in 1892; probably the first photo of the species (Gardeners' Chronicle 1892)

During the nineteenth century the botanical journals, gardens, fairs, and other horticultural events, contributed to the disclosure of this species (Fig. 5). Meanwhile, the seed and plant exchanges between gardens and the horticulturists actively contributed to the world distribution of this species (Table 4). More difficult to document is the possible plant transport linked with the slave trade and escaped slaves (Fleury 1994; Kull et al. 2015). Consequently, in the nineteenth century but also in the twentieth century this ornamental species was introduced throughout the world. Already, in the twenty-first century, the electronic commerce allows seeds selling all over the world without any indication of their invasiveness potential (e.g. Rare Exotic Seeds 2021; Fleurs des Tropiques 2021).

**Table 4:** Documented presence of *Hedychium gardnerianum* Sheph. ex Ker Gawl., Bot. Reg. 9: t. 774 (Feb. 1, 1824) as an ornamental plant in the nineteenth century

Presence on Botanical Garden			
** 1818**	**Cambridge Botanic Garden**	**England**	Lindley et al. (1823, 1826**)**
** 1819/1845**	**Calcutta Botanical Garden**	**India**	Roscoe (1828)** / **Voigt (1845)
1819	Liverpool Botanical Garden	England	Roscoe (1828)
** 1824**	**Royal Botanic Garden of Edinburgh**	**Scotland**	Graham (1825)
** 1824**	**Jardin Botanique de Fromont**	**France**	Bodin (1824)
1825/1892	Royal Botanical Garden of Glasgow	Scotland	Hooker (1825); Gardeners' Chronicle (1892)^a^
** 1828**	**Dresden Botanical Garden**	**Germany**	Felbel (1828)
1829	Jardin des Plantes (Paris)	France	Desfontaines (1829)
1831	Bonn Botanical Garden	Germany	Esenbeck & Sinning (1831)
1834/1842	Hamburg Botanical Garden	Germany	Lehmann (1834); MNHN (2021)
1836	Jardin de l'école de Médecine de Strasbourg	France	Fée (1836)
** 1838**	**Saint Petersburg Botanical Garden**	**Russia**	Fischer & Meyer (1838)
** 1845**	**Orto Botanico Di Napoli**	**Italy**	Tenore (1845)
1846	Brera Botanical Garden	Italy	Banfi & Visconti (2014)
** 1851**	**José do Canto Garden (S.Miguel Island)**	**Azores**	Canto (1851)
1852/1853	Horticultural Society's Garden (Chiswick)	England	Wallich (1853)
1854	Giardino del Conte de-Medici Spada	Italy	Amicucci (1854)
** 1858**	**Jardin Zoologique de Bruxelles**	**Belgium**	Galeotti (1858)
1859	Crystal Palace, London	England	Beaton (1859)
1865	António Borges Garden (S.Miguel Island)	Azores	Medeiros (1865)
** 1865**	**New Zealand Gardens**	**New Zealand**	Ludlam (1865)
1867	Iles du bois de Boulogne at Paris	France	Alphand (1867–1873)
** 1875**	**Queensland Botanic Gardens**	**Australia**	Hill (1875)
1875	Hatfield Gardens	England	Gardeners' Chronicle (1875)
1878	Botanical Garden of Coimbra University	Portugal	Henriques (1879)
1880	Conservatory at Buxted Park (Sussex)	England	Hogg (1880)
1883	Melbourne Botanic Gardens	Australia	Guilfoyle (1883)
** 1884**	**Santiago de Chile Botanical Garden**	**Chile**	Philippi (1884)
1885	Royal Botanic Gardens (Kew)	England	Hooker (1887)
1892	Mr. Conybear's Gardens (Tregullow)	England	Napper (1892)
1896	Joseph Chamberlain Garden (King’s Heath)	England	Watson (1896)
1896	Mr. Mark Rolle Gardens (Bicton, Devon)	England	Fraser (1896)
1898	Park of Pillnitz Castle (Dresden)	Germany	Rösner (1900)
Presence on exhibitiona events			
** 1830** /34/ 43/45/66	**Massachusetts Horticultural Society**	**U.S.A**	Fessenden (1831); Hovey (1834); Walker (1847); Strong et al. (1880)
1853	Société Royale Linnéenne de Bruxelles	Belgium	Galeotti (1853–54)
1875	Société d’Horticulture de Cherbourg	France	Angran (1876)
Presence on nurseries and seed/plant catalogues			
1830	Catalogue, New-York	U.S.A	Wm. R. Prince & Co. (1830)
1838 1839/42/58/ 60–61/63–66	Catalogue, St. Petersburg	Russia	Fischer & Meyer (1838); Fischer et al. (1839/42); Kuester et al. 1858); Regel & Herder (1860–61/63–66)
1854/71/72	Catalogue, Boston	U.S.A	Hoveys & Co. (1854/1871–72)
1854	Nursery, Planitz	Germany	Ender (1854)
1859	Nurseries, Belgium	Belgium	Funk (1859)
1866	Catalogue, Stuttgart	Germany	Pfitzer (1866)
1870	Nursery, Chelsea	England	Messrs. Veitch & Sons (1870)
1872	Nursery, Porto	Portugal	Junior (1872)
1885	Catalogue, New Jersey	U.S.A	Sturtevant (1885)
1897	Catalogue, Santa Barbara, Calfornia	U.S.A	Southern California Acclimatizing Association (1897)

In bold: the first documented presence in each nation/country

^a^First published photograph

## Escaping from cultivation

A revision of the literature and CABI, POWO, GISD and PIER databases (2021) allowed to update the world distribution and status of *H. gardnerianum* (Table 5).

**Table 5:** *Hedychium gardnerianum* distribution on the PIER, GISD, POWO and CABI databases and the present update proposal from this study

	PIER (2021)	GISD (2021)	POWO (2021)	CABI (2021a, )	This study	
Europe mainland						
Spain (Galicia)					INV	✓
Atlantic Islands						
Ascension			INT		INV	✓
Azores		INV	INT	INV	INV	
Canaries				INV	INV	
Cuba			INT		PI	✓
Dominican Republic/Haiti			INT		CUL	✓
Guadeloupe				INT	CUL	✓
Jamaica		INV	INT	INV	INV	
Madeira				INV	INV	
Martinique				INT	PI	✓
Tobago			INT		WR	✓
Trinidad			INT		ESC	✓
United Kingdom				INT	CUL	✓
Americas						
Brazil					INV	✓
Honduras			INT		ESC	
Mexico					INV	✓
Africa						
Kenya				NTS = Lapsus	CUL	✓
South Africa		INV		INV	INV	
Swaziland		INV			INV	
Zimbabwe					NTS	✓
Indian Ocean Islands						
Mauritius	INT		INT	INT	ESC	
Réunion	INV	INV	INT	INV	INV	
Rodrigues	INT			INT	WR	
Australia mainland						
New South Wales	INV		INT		INV	
Queensland	INV		INT	INV	INV	
South Australia					INV	✓
Victoria				INT	INV	
Pacific Ocean Islands						
Cook Islands	CUL	CUL	INT	INT	CUL	
Federated States of Micronesia	CUL	EST		INT	CUL	✓
Fiji	CUL			INT	INV	✓
French Polynesia	CUL	EST		INT	CUL	✓
Galapagos				INV = Lapsus	NP	✓
Hawaii	INV	INV	INT	INV	INV	
Japan			INT		CUL	
New Caledonia	CUL	CUL		INT	CUL	
New Zealand	INV	INV	INT	INV	INV	
Tasmania				INT	CUL	✓
Asia						
China	CUL				CUL	
Vietnam					ESC	✓

✓ Updated information; WR: Without reference; NP: Not present; INT: Introduced; CUL: Cultivated; EST Established; ESC: Escaped from cultivation; NTS: Naturalised; PI: Potentially Invasive; INV: Invasive. QLD: Queensland; NSW: New South Wales; VIC: Victoria; SA: South Australia

From all the French overseas communities *H. gardnerianum* is only considered invasive at Réunion (Soubeyran 2008). Although *H. gardnerianum* is mentioned as cultivated at Saint-Claude in 1897 (the Atlantic Guadeloupe Island) (Duss and Heckel 1897), the plant is not mentioned as escaped from cultivation in Flora de Guadeloupe (Stehlé 1935).

Concerning Rodrigues Island, this species is not mentioned in Botany of Rodrigues (Balfour 1879), Flora of Mascareignes—La Réunion, Maurice, Rodrigues (Antoine et al. 1983), and in Mascarine Cadetiana (2021) database.

Although *H. gardnerianum* is on the checklist of the vascular plants of Trinidad and Tobago (Baksh-Comeau et al. 2016), the island(s) where it occurs and the origin of that information are not specified: literature, herbarium specimen or field survey; for these reasons, we attain only to Caracciolo et al. (1892) where the species is referred as escaped from cultivation only on Trinidad Island.

Also, to both Rodrigues and Tobago islands, we were not able to find any reference of its use as a garden plant (Table 5).

In Japan (Ryukyu or Nansei Islands), the species is not mentioned in the book ‘Garden Plants of Japan’ (Levy-Yamamori and Taaffe 2004), but Tanaka et al. (2016) refers to a cultivar of *H. gardnerianum* cultivated on Koishikawa Botanical Gardens at Tokyo. The establishment of this species has not been confirmed and the Government of Japan (2012) puts *H. gardnerianum* only on a list of candidate species to be invasive on their islands (Table 5).

*H. gardnerianum* is not present at Galapagos (the species escaped from cultivation at Galapagos is *H. coronarium*) (CABI 2021b) (Table 5) and is under cultivation in the United Kingdom (Fig. 2k), Dominican Republic/Haiti (Maas and Maas 1990), Guadeloupe (Duss and Heckel 1897; Fournet 2002), Kenya (Witt and Luke 2017), Tasmania (PAHSMA 2014; AVH 2021), New Caledonia (Grande Terre) (MacKee 1994; Hequet et al. 2009), Cook Islands (Rarotonga and Mangaia) (Space and Flynn 2002; McCormack 2013), French Polynesia (Tahiti) (Florence et al. 2013), Federated States of Micronesia (Pohnpei) (Fosberg et al. 1987; Herrera et al. 2010), and China (Wu and Raven 2000) (Table 5).

References, dry specimens and/or photos support that *H. gardnerianum* had or is escaped from cultivation at Galicia (Spain), Azores archipelago, Madeira Island, Tenerife Island, Cuba, Jamaica, Martinique, Trinidad, Ascension, Mexico, Honduras, Brazil, South Africa, Swaziland, Zimbabwe, Réunion, Mauritius, Australia (QLD, NSW, VIC, SA) New Zealand, Fiji, Hawaii, and Vietnam (Table 6). We found references of the presence of this species in the nineteenth century (cultivated or escaped) in all of those regions with exception of Cuba, Mexico, Honduras, Ascension, South Africa, Swaziland, and Zimbabwe (Table 6).

**Table 6:** Documented dates and regions where *Hedychium gardnerianum* was mentioned as cultivated or escaped from cultivation [Specimen number]. Lines within each region ordered by the year of column: Escaped from cultivation

Region	Not mentioned		Cultivated		Escaped from cultivation	
	Year	Reference	Year	Reference	Year	Reference
Europe						
Spain						
Galicia: Muros and peninsula del Morrazo					2000	Silva-Pando et al. (2009)
Atlantic Islands						
*Trinidad*						
	1864	Grisebach (1864)			1892	Caracciolo et al. (1892)
	1869	Prestoe (1869)			2016	Baksh-Comeau et al. (2016)
	2020	Government of Trinidad and Tobago (2021)				
*Jamaica*						
	1864	Grisebach (1864)			1893	Fawcett (1893)
Cinchona					1913	Goodland & Healey (1996)
*Madeira*						
Madeira Island			1832-55^a^	Lowe (1857)		
São Vicente					1894	Menezes (1894)
Funchal Gardens			1914	Menezes (1914)		
*Azores*						
S. Miguel	1847	Canto (1847)	1851	Canto (1851)	1894–96	Trelease (1897)
Flores					1894–96	Trelease (1897)
*Ascension*						
	1836	Darwin (1845)				
	1851	Seemann (1852–1857)				
					2004	Fairhurst (2004)
Elliots path					2020	Croson (2020)
*Cuba*						
	1989	Esquivel et al. (1989)	2003	Shagarodsky (2003)		
Parque Nacional La Bayamesa					2005	Sanchez-Ruiz (2005)
			2008	De Zayas (2008)	2011	Gonzaléz et al. (2012)
*Canaries*						
Tenerife Island						
Orotava			1893	Wilks & Weathers (1896)		
Orotava			1923	Menéndez (1923)		
Parque García Sanabria			1990	Rodriguez (1990)		
Parque García Sanabria			2000	Reyes Y Pérez (2001)		
Rural Park of Anaga					2011	Dela Rosa et al. (2014)
*Martinique*						
Surroundings of Saint Pierre [P01674257]			1853 Reflora (2021) (probably cultivated)			
Americas						
*Brazil*						
Minas Gerais						
Ouro Preto [MO1344768]					1895	Reflora (2021)
Universidade Federal de Juiz de Fora					2018	Tavares-Silva et al. (2018)
Paraná						
Antonina (Specimen 32.755)					1974	I^3^N Brasil (2021)
Tijucas do Sul, Represa do Vossoroca (Guaratuba Environmental Protection Area)					2004	Blum et al. (2005)
Guaraqueçaba					2008	I^3^N Brasil (2021)
São Paulo						
Municipal Natural Park of Cratera de Colônia					2009	Marçon (2009); I^3^N Brasil (2021)
Rio de Janeiro			1890	Martius et al. (1890)		
Andarai Grande [P01674255]			1871	Reflora (2021)		
Tijuca [R010065496]			1886	Reflora (2021)		
Jardim Botânico			1937	Penna (1937)		
Itatiaia National Park					2013	I^3^N Brasil (2021)
Santa Catarina						
São Bento do Sul					2019	Meyer and Schwirkowski (2019)
*Mexico*						
Chiapas (Cerro Ovando)					1950	Matuda (1950)
Chiapas (Mapastepec)					2005–07	Ayuntamiento Municipal (2005/07)
Oaxaca					2019	Dirección General de Impacto y Riesgo Ambiental (2019)
Campeche					2011	González-Lazo (2011)
*Honduras*						
			1975	Molina (1975)	2008	Sutherland (2008)
Africa						
*South Africa*						
	1898	Baker (1898)				
	1991	Macdonald (1991)				
Kwazulu-Natal			1894^b^	Wood (1895)	1998	Smith (1998); Henderson (2001)
Kruger National Park (Mpumalanga and Limpopo)					1999	Foxcroft et al. (2003); Henderson (2001)
*Zimbabwe*						
Vumba Botanic Garden					2004	Hyde et al. (2021)
Harare					2006/2020	Hyde et al. (2021)
Juliasdale					2014	Hyde et al. (2021)
Chimanimani					2015	Hyde et al. (2021)
*Swaziland*						
	1998	Smith (1998)			2021	Swaziland's Alien Plants Database (2021)
Mlilwane Wildlife Sanctuary/islanddweller					2021	BioDiversity^4^All (2021)
Indian Ocean Islands						
*Réunion Island*						
Saint Denis Botanical Garden	1820/25	Breon (1820, 1825)				
Saint Denis Botanical Garden	1825	Breon (1825)				
Saint Denis Botanical Garden	1856	Richard (1856)				
			1910/11	Commissioner of Agriculture for the West Indies (1912)	1895	Cordemoy (1895)
*Mauritius*						
	1877	Baker (1877)			2021	Mascarine Cadetiana (2021)
Rivière Noir			1837^c^	Bojer (1837)		
Australia						
Queensland, Brisbane			1875	Hill (1875)		
Victoria, Melbourne			1883	Guilfoyle (1883)		
New South Wales, Morisset [NSW 687345]			1920	AVH (2021)		
New South Wales, Ingleburn					1968	Csurhes & Hannan-Jones (2016)
South Australia [AD 281835]					2018	AVH (2021)
Pacific Ocean Islands						
*New Zealand*						
	1841	Hooker (1853)	1865	Ludlam (1865)		
North Island					1940	Allan (1940)
South Island [CHR: 81,349, 481,420; 326,334 A, HO538398]					1953/75/77/86	AVH (2021)d
Auckland					1949	Williams et al. (2003)
Chatham Island [AK307272]			Before 2009, AVH (2021) (cultivated or escaped)			
*Hawai’i*						
	1934	Degener (1934)				
Hawai’i Volcanoes National Park (Park residential area)					1947	Fagerlund (1947)
Hawai’i					1954	Stone et al. (1992)
O’ahu					1975	Stone et al. (1992)
All islands					1985	Smith (1985)
*Fiji*						
Viti Levu			1979	Smith (1979)		
Viti Levu: Nukunuku, Nadala and Qaliwana Creeks					2002/04	Boseto (2006)
Asia						
*Vietnam*						
Ho Chi Minh (Saigon) [CLF082528]			1909	IHU (2021)		
					1993	Ho (1993)
Cat Ba national park					2012	Tan et al. (2012)

^a^*Hedychium* sp.; ^b^*Hedychium* sp. from East Indies; ^c^Under *H. speciosum* name

Concerning Brazil, in 1890 Martius et al. states that *H. gardnerianum* is cultivated at Rio de Janeiro and indicates the specimen collected by Glaziou in 1871 (Reflora 2021). In another specimen label collected in 1895 (Reflora 2021) we can read: ‘arbusto silvestre de solo úmido fl vermelhadas aromáticas’ (wet soil wild shrub with aromatic reddish flowers), pointing out its spontaneous occurrence in a suitable habitat.

At Mexico, Matuda (1950) found *H. gardnerianum* in favourable conditions at 1000–1500 m altitude in a wet ravine of Cerro Ovando (Chiapas). Although no recent botanic studies were made to prove the spreading of this species, the species is mentioned in 3 environmental impact assessments at Mapastepec (Chiapas) and the nearby states of Oaxaca and Campeche (Ayuntamiento Municipal de Mapastepec 2005/07; Dirección General de Impacto y Riesgo Ambiental 2019; González-Lazo 2011).

*H. gardnerianum* is also a well-known flower in the island of Ascension (The Islander 2003), that appears on a stamp collection of wild flowers in 1985 (Colnect 2021) and in the field guide of Fairhurst (2004). Although it is not mentioned in a botanical survey (Lambdon and Darlow 2008) or listed in Pagad and Wong (2020) database, a picture taken at 26^th^ June 2020 at Elliots path by Croson (2020), supports the hypothesis that the species is naturalized.

At Zimbabwe, *H. gardnerianum* escaped from cultivation at Vumba, Juliasdale, Chimanimani, and Harare (Hyde et al. 2021). In Chatham Island (New Zealand) a plant specimen (HO521919) was harvested but is not referred if it was a cultivated or a spontaneous plant (AVH, 2021).

To Viti Levu Island (Fiji) a 2006 Master thesis (Boseto 2006) about freshwater fishes identifies *H. gardnerianum* escaped from cultivation near three creeks, but it remains to explain the source and the reason of this recent escape.

*H. gardnerianum* is also a recent escape with invasive characteristics in European mainland (Spain, Galicia) with no reference of the putative source of this escape (Silva-Pando et al. 2009).

Regarding the regions where the species escaped from cultivation, we looked for the first references to its cultivation (Table 6) and according to the literature we classified the severity of these escapes in: escaped from cultivation; escaped from cultivation and potentially invasive; Invasive process started; Invasive process established (Table 7).

**Table 7:** Present status of invasiveness severity in the regions where the species escaped from cultivation according literature

Region	Severiry	Reference	
	Classification	Classification by the authors	In:
Europe			
Spain (Galicia)	3	Moderate	Ramil-Rego and Vales (2019)
Atlantic Islands			
Ascension	3	Present (Flora Guide)	Fairhurst (2004)
Azores	4	Invasive	Silva et al. (2008)
Canaries (Tenerife)	3	Initial state of dispersion	De La Rosa et al. (2014)
Cuba	2	Potentially invasive	González-Torres et al. (2012)
Jamaica	4	Invasive	Iremonger (2002)
Madeira	4	Invasive	Silva et al. (2008)
Martinique	2	Naturalized around the gardens	UICN|Comité Francais (2017a, b)
Trinidad	1	Present (Flora Checklist)	Baksh-Comeau (et al. 2016)
Americas			
Brazil	4	Invasive	I^3^N Brasil (2021)
Honduras	1	Present (Flora checklist)	Sutherland (2008)
Mexico (Mt. Ovando)	3	Invasive	Matuda (1950)
Africa			
South Africa	4	Emerging invader	Nel et al. (2004)
Swaziland	3	Invasive, potential problem species	Swaziland's Alien Plants Database (2021)
Zimbabwe	3	Naturalized	Hyde et al. (2021)
Indian Ocean Islands			
Réunion	4	Invasive	UICN Comité Francais (2017a, b)
Mauritius	1	Present	Mascarine Cadetiana (2021)
Australia			
New South Wales Queensland South Australia Victoria	4	Naturalised in New South Wales and adventive in Victoria; Potential range Queensland and Western Australia	Csurhes & Hannan-Jones (2016)
Pacific Ocean Islands			
Fiji	3	Spontaneous on creeks	Boseto (2006)
Hawaii	4	Invasive	Minden et al. (2010b)
New Zealand	4	Environmental weed; Pest plant	Howell (2008); New Zealand Government (2020)
Asia			
Vietnam	1	Insignificant	Tan et al. (2012)

The species *status* in each region is here classified as: 1. Escaped from cultivation; 2. Escaped from cultivation and potentially invasive; 3. Invasive process started; 4. Invasive process established

**Fig. 6:**
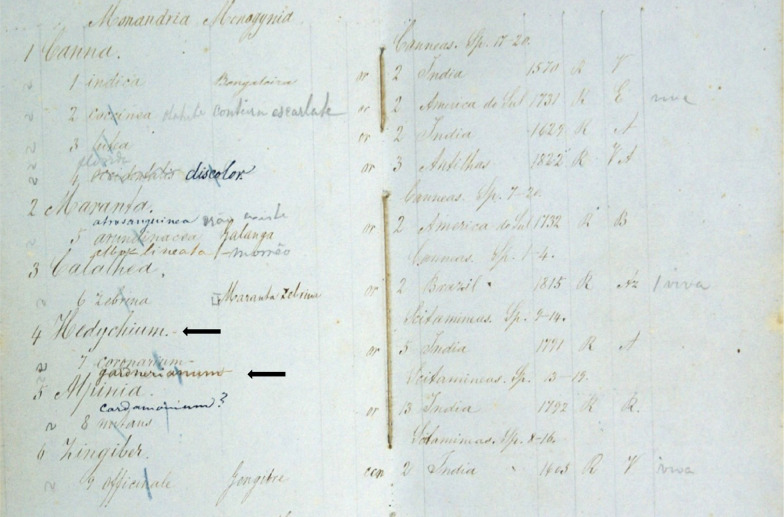
Manuscript list of the cultivated plants in José do Canto Garden, in the year 1851 (S. Miguel Island, Azores). *Hedychium gardnerianum* () (Courtesy of Azores University Library)

Although it is historically and environmentally important to know the year of introduction of an invasive species, this knowledge is often missing (Table 6). Regarding the Azorean Islands we found in a recent bequest to the Azores University Library (still not inventoried), a manuscript list of the plants cultivated at Ponta Delgada made by José do Canto in 1847 where *H. gardnerianum* is not listed, while in 1851 the species is already cultivated at S. Miguel Island according to the same gentleman farmer (Canto 1851) (Fig. 6). *H. gardnerianum* is also present in another manuscript list of cultivated plants at Ponta Delgada written by António Borges da Câmara de Medeiros in 1865 (António Borges Garden), found at Ponta Delgada Public Library and Regional Archive. Other nineteenth century documents available online allowed to verify the presence of this species as an ornamental plant at Canaries (1893), Martinique (1882), Brazil (1871), Australia (1875/83) and New Zealand (1865) (Table 6). Regarding the islands of Madeira, Jamaica and Réunion, where the species escaped from cultivation, we still did not find a reference of its first presence as an ornamental plant in the nineteenth century. In Madeira, only the genus is referred to be under cultivation (Lowe 1857). Concerning Jamaica, a reference of the species being cultivated at Chinchona Botanical gardens after its constitution (1868) and before becoming naturalized (1893) is still missing (Goodland and Healey 1996). The analysis of the following references, Bellingham et al. (2005) and Grubb and Tanner (1976), did not allow to confirm the year of introduction pointed out by Hulme (2011). Finally to the Réunion island we could not find any reference to support the year of introduction. In 1817 the botanical garden of Réunion Island (Jardin du Roi at Saint-Denis) receives the first plants from Europe and in 1820 it's Director Nicolas Bréon acknowledges the gifts sent by several personalities including N. Wallich, but in its catalogue, the only cultivated species of *Hedychium* is *H. coronarium*; in its 1825 catalogue two more *Hedychium* species are cultivated: *H. ellipticum* and *H. flavescens.* In 1856, the next Director of this garden, Jean-Michel Claude Richard, also publishes a catalogue with the cultivated plants, where no species of *Hedychium* is mentioned. Nevertheless, due to the Malagasy-origin creole name (Creole name: longouze with longoza as Malagasy root), it is possible that slaves and marooned slaves had their role in this species propagation at Réunion; in fact, *Hedychium* spp. are identified among the food plants of marooned slaves from East Africa, Madagascar, and the Mascarene Islands (Kull et al. 2015). Lastly, Cordemoy (1895) only states that *H. gardnerianum* is abundantly naturalized with no mention of the year of introduction.

Regarding the severity of these escapes (Table 7) to Mauritius, Honduras, and Trinidad there is little information about the presence and abundance of this species and fieldwork is needed to confirm the species current status; to Vietnam the risk is considered insignificant; to Martinique the species is naturalized around gardens, and at Cuba is considered only potentially invasive. At Galicia, Canaries, Mexico, Ascension, Swaziland, Zimbabwe, and Fiji the invasive process already started but the severity of its progress would be modulated by the environment and control actions on the field. The invasive process is well established at Azores, Madeira, Jamaica, Réunion, New Zealand and Hawaii and the same in some countries of Central and South America, Southern Africa, and Mainland Australia, but in these mainland areas the species still continues to expand its distribution area.

## Conclusions

This research was only possible due to the valuable resources already available online, namely, the Herbaria digitalized specimens or the digitalized historical documents at Biodiversity Heritage Library and Internet Archive. However, although the word search tool is extremely useful to accelerate the research inside documents, in some situations the names are not detected, e.g. a lack of a letter due to digitalization quality, letters not perfectly printed or misspelt words (e.g. Fessenden 1831). Also, this tool cannot be used in handwritten or gothic script documents slowing the research process. A conjoint effort between linguistic, historic, and botanic fields would also improve the access and interpretation of old documents and documents in different idioms.

In synthesis, this study updated the information about *H. gardnerianum* scientific discovery, nomenclature, types, native range and regions where it is considered escaped from cultivation and the severity of these escapes. This research found some new information as the introduction of *H. gardnerianum* in José do Canto Garden after 1847 and before 1851 and identified new information with interest to plant data bases as the escapes from cultivation of *H. gardnerianum* at Viti Levu and Mexico, or with historic interest as the reference of the presence of *H. gardnerianum* since 1818 at Cambridge Botanical Garden. This study also clarified some aspects of its native range as the exclusion of the present Bangladesh as a natural area of the species distribution; detected and clarified same lapsus at the data bases about the years of introduction at Réunion and Jamaica (still not known) or about the species distribution (Galapagos and Kenya) or even about the name authority. Moreover lacks of information were identified as the years of introduction of the species (e.g. who send the seeds or rhizomes to the Cambridge Botanical Garden?), or about the severity of its escapes in certain regions. Although *Hedychium* spp. are cultivated worldwide, there is a substantial paucity of studies about the presence and spreading of this species in Central and South America, Africa and several oceanic islands. Finally, *H. gardnerianum* is a serious pest in Azores, Madeira, Jamaica, Réunion, New Zealand and Hawaii and continues to expand its distribution area in South and Central America, Australia and Southern Africa. The species continues to escape from cultivation as the recents escapes in Tenerife and Viti Levu islands and Galicia.

While in some regions two or three species of the genus are considered invasive (e.g. Brazil) in others although two species are considered escaped from cultivation one wined the invasive status above the other (e.g. Azores); a future analysis of expansion risk of this species should consider all the *Hedychium* spp. too. The same for several specially frequent trouble associations as *H. gardnerianum* plus *Pittosporum undulatum* (e.g. in Azores, Jamaica and Hawaii).

In summary, the analysis of the available information allowed to conclude that: (a) *Hedychium gardnerianum* is a validly published name, the authority of the name is Sheph. ex Ker Gawl., the species holotype is the illustration published along with the species name, and the Natural History Museum BM000574691 specimen collected in 1815 is the first dried specimen of *H. gardnerianum*; (b) This species is native to the Central and Eastern Nepal, Bhutan, Northeast India and North Myanmar; (c) The species was cultivated at Cambridge Botanical Garden since 1818 and the first known herbarium specimen collected in Europe dates back to 1821; (d) Kathmandu (Nepal) and Khasi Hills (India) specimens are considered two varieties of the same species and the BM000574691 specimen is the lectotype of *H. gardnerianum* var. *speciosum*; (e) Specimens, references, and/or pictures support that *H. gardnerianum* escaped from cultivation at Galicia (Spain), Azores archipelago, Madeira, Tenerife, Cuba, Jamaica, Martinique, Trinidad, Ascension, Mexico, Honduras, Brazil, South Africa, Swaziland, Zimbabwe, Réunion, Mauritius, Australia, New Zealand, Fiji, Hawaii, and Vietnam; and (f) *H. gardnerianum* is a serious pest in Azores, Madeira, Jamaica, Réunion, New Zealand and Hawaii and continues to expand its distribution area in South and Central America, Australia and Southern Africa.

## Data Availability

All sources of information are are included in this published article. All data generated or analysed during this study are included in this published article.

## Abbreviations

CABI: Centre for Agriculture and Bioscience International
e.g.: exempli gratia
et al.: et alii
GISD.: Global Invasive Species Database
H.: *Hedychium*
IPNI: International Plant Names Index
NSW: New South Wales
PIER: Pacific Island Ecosystems at Risk
POWO: Plants of the World Online
QLD: Queensland
SA: South Australia
UK: United Kingdom
var.: variety
VIC: Victoria

## References

[CR1] Alien Invasive Plants List for South Africa (2021) Weeds, invaders and alien vegetation. https://www.environment.co.za/weeds-invaders-alien-vegetation/alien-invasive-plants-list-for-south-africa.html. Accessed 21 Jun 2021.

[CR2] Allan H (1940) A handbook of the naturalized flora of New Zealand. Department of Scientific and Industrial Research - Bulletin 83, New Zealand.

[CR3] Alphand A (1867–1873) Les promenades de Paris: histoire, description des embellissements, dépenses de création et d'entretien des Bois de Boulogne et de Vincennes, Champs-Elysées, parcs, squares, boulevards, places plantées, études sur l'art des jardins et arboretum. J. Rothschild Éditeur, Paris.

[CR4] Amicucci R (1854) Giardino del Conte de-Medici, Spada a Villa Quiete, presso Treja nelle Marche. Della Tipografia Bianchini, Macerata.

[CR5] Angran M (1876). Rapport sur la 21^e^ Exposition de la Société d’Horticulture de Cherbourg. Bulletin de la Société d’Horticulture de Cherbourg, 7e Anné. Année.

[CR6] Antoine R, Brenan JPM, Mangenot G (1983) Flora of Mascareignes: La Rénion, Maurice, Rodrigues: 171. Zingibéracées à 176. Bromeliacées. The Sugar Industry Research Institute, Mauritius; L'Office de la Recherche Scientifique et Technique Outre-Mer, Paris; The Royal Botanic Gardens, Kew.

[CR7] Arruda M, Viana H, Rainha N, Neng NR, Nogueira JM, Barreto MC (2012). Anti-acetylcholinesterase and antioxidant activity of essential oils from *Hedychium gardnerianum*. Molecules.

[CR8] Asner GP, Vitousek PM (2005). Remote analysis of biological invasion and biogeochemical change. PNAS.

[CR9] Atlas of Living Australia (2021) Open access to Australia’s biodiversity data. https://www.ala.org.au/. Accessed 21 Jun 2021.

[CR10] AVH (2021) The Australasian Virtual Herbarium. https://avh.chah.org.au/. Accessed 21 Jun 2021.

[CR11] Ayuntamiento Municipal de Mapastepec, Chiapas (2005–2007) Manifiesto de Impacto Ambiental: Resumen Ejecutivo. Estados Unidos Mexicanos. http://sinat.semarnat.gob.mx/dgiraDocs/documentos/chis/resumenes/2007/07CH2007HD049.pdf. Accessed 21 Jun 2021.

[CR13] Baker JG (1877) Flora of Mauritius and the Seychelles: a description of the flowering plants and ferns of those islands. Colonial Government of Mauritius (ed), L. Reeve & Co., London.

[CR12] Baker JG, Thiselton-Dyer WT (1898). Scitaminae. Flora of tropical Africa, vol 7.

[CR14] Baksh-Comeau Y, Maharaj SS, Adams CD, Harris SA, Filer DL, Hawthorne WD (2016). An annotated checklist of the vascular plants of Trinidad and Tobago with analysis of vegetation types and botanical 'hotspots'. Phytotaxa.

[CR15] Balfour IB (1879). Botany of Rodriguez. Philos Trans R Soc Lond B..

[CR16] Banfi E, Visconti A (1846). The history of the botanic garden of Brera during the restoration of the Austrian empire and the early years of the kingdom of Italy. Nat Hist Sci..

[CR17] Barcode of Life Data System v4 (2021) Advancing biodiversity science through DNA-based species identification. http://www.barcodinglife.org/index.php/. Accessed 21 Jun 2021.

[CR18] Baret S, Rouget M, Richardson DM, Lavergne C, Egoh B, Dupont J, Strasberg D (2006). Current distribution and potential extent of the most invasive alien plant species on La Réunion (Indian Ocean, Mascarene islands). Austral Ecol.

[CR19] Baruah MK, Choudhury MD (2014). Addition to the Flora of Barak Valley of Assam. India Pleione.

[CR20] Basak S, Ramesh AM, Kesari V, Parida A, Mitra S, Rangan L (2014). Genetic diversity and relationship of *Hedychium* from Northeast India as dissected using PCA analysis and hierarchical clustering. Meta Gene.

[CR21] Beaton D (1859). Crystal Palace - January 11. The Cottage Gardener.

[CR22] Bellingham PJ, Tanner EVJ, Healey JR (2005). Hurricane disturbance accelerates invasion by the alien tree *Pittosporum undulatum* in Jamaican montane rain forests. J Veg Sci.

[CR23] Bernhardi JJ, Völker HLW (1825) Luftgårtnerei, 774 *Hedychium gardnerianum*. Neues allgemeines Garten-Magazin :160.

[CR24] Bilder aus Nepal (2021) Edward Gardner, British Resident 1816–1829. https://www.bilder-aus-nepal.de/Pages/Geschichte/Edward-Gardner*.html. Accessed 21 Jun 2021.

[CR25] Biodiversity Heritage Library (2021) Inspiring discovery through free access to biodiversity knowledge. https://www.biodiversitylibrary.org/. Accessed 21 Jun 2021.

[CR26] BioDiversity4All (2021) January 27, 2020, Mlilwane Wildlife Sanctuary, Islanddweller. https://www.biodiversity4all.org/observations/38287691. Accessed 21 Jun 2021.

[CR27] Blum CT, Posonski M, Hoffmann PM, Borgo M (2005) Espécies vegetais invasoras em comunidades florestais nativas nas margens da Represa do Vossoroca, APA de Guaratuba, Paraná, Brasil. In: Ministério do Meio Ambiente (ed.) I Simpósio Brasileiro sobre Espécies Exóticas Invasoras, Brasília, 2005.

[CR28] Bodin MS (1824). Récit d’une excursion horticulturale faite à Londres dans le mois d’avril. Annales De L'agriculture Française.

[CR29] Bojer W (1837). Hortus Mauritianus.

[CR30] Bose MAJC (2015–2016) Gingers of Nagaland. In: Sinha BK, Dash SS, Pramanick DD, Kumar SS (eds) Botanical survey of India Annual Report 2015–2016. Kolkata: Ministry of Environment Forest & Climate Change. 124.

[CR31] Boseto D (2006) Fiji diversity, distribution, and abundance of Fijian freshwater fishes. Master thesis, University of South Pacific.

[CR32] Breon N (1820) Catalogue des plantes cultivées aux jardins botanique et de naturalisation de l'ile Bourbon. L’Imprimerie du Gouvernement, Saint-Denis.

[CR33] Breon N (1825) Catalogue des plantes cultivées aux jardins botanique et de naturalisation de l'ile Bourbon. L’Imprimerie du Gouvernement, Saint-Denis.

[CR34] Brongniart MAd (1824). Botanique 309 Botanical Register no108 février 1824. Bulletin Des Sciences Naturelles Et De Geologie.

[CR35] CABI (2021a) *Hedychium gardnerianum* [D Djeddour] In: Invasive Species Compendium. CABI, Wallingford. https://www.cabi.org/isc/datasheet/26679. Accessed 21 Jun 2021.

[CR36] CABI (2021b) *Hedychium coronarium* [D Djeddour, Ja Rojas-Sandoval, P Acevedo-Rodríguez] In: Invasive Species Compendium. CABI, Wallingford. https://www.cabi.org/isc/datasheet/26678. Accessed 21 Jun 2021.

[CR37] Cable J (2017) Flowering *Hedychium gardnerianum* in summer of 2017 at Walled Garden in Croxteth Hall and Country Park. In: Dina Younis Blog (25 Oct 2017) about the practical training ‘Work and retrain as a Gardener Scheme’ run by the ‘Women’s Farm and Garden Association’. https://croxtethhallwrags.wordpress.com. Accessed 21 Jun 2021.

[CR38] Canto J (1847) Catálogo das plantas existentes em Stª Anna com etiquetas de chumbo por extenso começado em Janeiro de 1847. UACSD / FAM-ABS-JC / Non-inventoried documentation (Nestor de Sousa bequest).

[CR39] Canto J (1851) Hortus Cantuanus. Relação das plantas cultivadas na ilha de S. Miguel por José do Canto no anno de 1851. UACSD / FAM-ABS-JC / Untreated documentation / Box. 156.

[CR40] Caracciolo H, Cardomy P, Devenish S, Mole RR, Urich FW (1892). Report of Club Meetings, 4th November. J Field Nat Club.

[CR41] Carey W (ed) (1820) Flora Indica or Descriptions of Indian Plants, by the Late William Roxburgh. Vol.I. Edited by William Carey, D.D., to which are added descriptions of plants more recently discovered by Nathaniel Wallich. Serampore, West Bengal.

[CR42] Catalogue of Life (2021) Authoritative list of the world's species. https://www.catalogueoflife.org/col/search/all. Accessed 21 Jun 2021.

[CR43] Chauchard S, Lavergne C (2009) Suivi de l’impact de la lutte contre les espèces exotiques envahissantes à la Réunion: le cas du longose (*Hedychium gardnerianum* Sheppard ex Ker Gawl.) sur le site des Makes. Rapport technique N°6, Conservatoire Botanique National de Mascarin, Saint-Leu, Réunion.

[CR44] Colnect (2021) Stamps, Ascension Island, Collection Wildflowers, *Hedychium gardnerianum.* https://colnect.com/en/stamps/stamp/325486-Ginger_Lily-Wild_Flowers-Ascension_Island. Accessed 21 Jun 2021.

[CR45] Commissioner of Agriculture for the West Indies (1912) Gleanings: From Diplomatic and Consular Reports No. 4865, Annual Series, The Agricultural News, 11(265): 204.

[CR46] Cordemoy J (1895). Flore de l'île de La Réunion (Phanérogames, Cryptogames vasculaires, Muscinées).

[CR47] Croson S (2020). 26 June 2020 Picture of *Hedychium gardnerianum* at Elliot path. https://www.facebook.com/AscensionIslandConservation/. Accessed 21 Jun 2021.

[CR48] Csurhes S, Hannan-Jones M (2016) Invasive plant risk assessment; Kahili ginger *Hedychium gardnerianum*, White ginger *Hedychium coronarium*, Yellow ginger *Hedychium flavescens*. Department of Agriculture and Fisheries Biosecurity-Queensland Government, Queensland

[CR49] Daimei P, Kumar Y (2011). Occurrence of *Hedychium* Koenig (*Zingiberaceae*) in Tamenglong District of Manipur. Northeast India Pleione.

[CR50] Darwin C (1845). Journal of researches into the geology and natural history of the countries visited during the voyage of HMS Beagle round the world.

[CR51] Das Gupta U, Chattopadhyaya DP (2011). Science and modern India: An Institutional History, c 1784–1947. Series History of Science Philosophy and Culture in Indian Civilization Volume XV Part 4.

[CR52] De La Rosa A, Martín VE, Wildpret W (2014). *Hedychium gardnerianum* Sheppard ex Ker Gawl (*Zingiberaceae*), nueva especie invasora en las islas Canarias. Vieraea.

[CR53] de Penna L (1937). Notulas botânicas: Floração de Outono. Rodriguésia.

[CR54] De Zayas A (2008) Plantas ornamentales en Cuba: Usos, diversidad y amenazas. Revista Del Jardín Botánico Nacional, 29: 83–100. http://www.jstor.org/stable/42597271. Accessed 21 Jun 2021.

[CR55] Degener O (1934) Flora Hawaiiensis or New illustrated flora of the Hawaiian Islands. Fl. fam. 76. *Hedychium coronarium*. 10/15/’34 *Hedychium flavum*. 10/12/’34. Publ. privately, Honolulu, Hawaii.

[CR56] Desfontaines R (1829) Catalogus Plantarum Horti regii parisiensis cum annotationibus de plantis novis aut minus cognitis. 3rd edn. Chaudé J.S. Ed., Parisiis.

[CR57] Dirección General de Impacto y Riesgo Ambiental (2019) Manifestación de Impacto Ambiental Modalidad Regional: Construcción de un camino tipo “E” Santiago Yaitepec - Santa María Temaxcaltepec del Km 0+000 – Km 20+000, en el Edo de Oaxaca. Estados Unidos Mexicanos. https://apps1.semarnat.gob.mx:8443/dgiraDocs/documentos/oax/estudios/2019/20OA2019V0072.pdf. Accessed 21 Jun 2021.

[CR58] Duss RP, Heckel E (1897). Flore phanérogamique des Antilles françaises (Guadeloupe et Martinique). Annales De L'institut Colonial De Marseille.

[CR59] Eleutério T, Pinto AS, Pereira MJ, Vasconcelos HC (2017). Preliminary structural and thermal characterization of conteira’s (*Hedychium gardnerianum*) fibres for further functionalization with silica colloidal nanoparticles. Proc Eng.

[CR60] Eleutério T, Pereira MJ, Vasconcelos HC (2018). Effect of extraction method on physicochemical characteristics of kahili ginger (*Hedychium gardnerianum*) fibres. Mater Sci Nanotechnol.

[CR61] Eleutério T, Sério S, Teodoro OMND, Bundaleski N, Vasconcelos HC (2020). XPS and FTIR studies of DC reative magnatron sputterd TiO2 thin films on natural based-cellulose fibers. Coatings.

[CR62] Encyclopedia of Life (2021) Global access to knowledge about life on Earth. https://eol.org/pages/1118152. Accessed 21 Jun 2021.

[CR63] Ender E (1854). Geitner’s Treibegärtnerei zu Planitz. Österreichisches botanisches Wochenblatt gemeinnütziges Organ für Botanik und Botaniker, Gärtner, Oekonomen, Forstmänner, Aerzte. Apothekeru Techniker.

[CR64] Esenbeck TFNL, Sinning W (1831). Sammlung schönblühender gewächse für blumen-und gartenfreunde nach lebenden exemplaren des Königlichen botanischen gartens zu Bonn gezeichnet beschrieben und mit genauer anleitung zu ihrer cultur begleitet.

[CR65] Esquivel M, Castiñeiras L, Knopffer H, Hammer K (1989). A checklist of the cultivated plants of Cuba. Kulturpflanze.

[CR66] European Vegetation Archive (2021) Vegetation of Europe. https://www.synbiosys.alterra.nl/evc/. Accessed 21 Jun 2021.

[CR67] Fagerlund GO (1947) The exotic plants of Hawaii National Park. Nat. Hist. Bull. 10, Hawaii National Park.

[CR68] Fairhurst W (2004). Flowering Plants of Ascension Island.

[CR69] Fawcett W (1893). A provisional list of the indigenous and naturalized flowering plants of Jamaica.

[CR70] Fée A (1836) Catalogue méthodique des plantes du Jardin de l'école de Médecine de Strasbourg. Imprimerie F.G. Levraut, Strasbourg.

[CR71] Felbel (1828) Berzeichß von Warm und Kalt Hauspflanzen welche in Dresden bei dem gärtner Felbel. Neues allgemeines Garten-Magazin: XVII-XXII.

[CR72] Fessenden T (ed) (1831) Massachusetts Horticultural Society - Flowers, Saturday, August 14, 1830. The New England Farmer and Horticultural Journal, August 20, 1830:38.

[CR73] Fischer FEL, Meyer CA (1838) Index Seminum, quae Hortus Botanicus Imperialis Petropolitanus pro mutua commutatione offert, Accedunt animadversiones botanicae nonnullae. Petropoli.

[CR74] Fischer FEL, Meyer CA, Avé-Lallemant JLE (1839/42) Index Seminum, quae Hortus Botanicus Imperialis Petropolitanus pro mutua commutatione offert, Accedunt animadversiones botanicae nonnullae. Petropoli

[CR75] Fleurs des Tropiques (2021) *Hedychium gardnerianum* / Longose - lot de 20 graines - 2.25€. https://www.fleurdestropiques.net/hedychium-gardnerianum-longose-lot-de-20-graines-c2x2422190. Accessed 21 Jun 2021.

[CR76] Fleury M (1994). Impact de la traite des esclaves sur la phytogéographie: exemple chez les Aluku (Boni) de Guyane française. J D'agriculture Traditionnelle Et De Botanique Appliquée.

[CR77] Flora-on Açores (2021) *Hedychium gardnerianum*. https://acores.flora-on.pt/#/1hedychium+gardnerianum. Accessed 21 Jun 2021.

[CR78] Flora of Nepal (2021) *Hedychium gardnerianum* - Adhikari B. Parmar G. Pandey T. Chhetri R. & Amrit K. 80 – Photographer Adhikari B. – Observed in Nepal Province 3 Lalitpur District. http://www.floraofnepal.org/imagegallery. Accessed 21 Jun 2021.

[CR79] Flora of New Zealand (2021) The definitive reference to New Zealand plants. http://www.nzflora.info/factsheet/Taxon/Hedychium-gardnerianum.html. Accessed 21 Jun 2021.

[CR80] Florence J, Chevillotte H, Ollier C, Meyer JY (2013) Base de données botaniques Nadeaud de l'Herbier de la Polynésie Française (PAP). http://archive.is/GQKMc. Accessed 21 Jun 2021.

[CR81] Fosberg FR, Sachet MH, Oliver R (1987). A geographical checklist of the Micronesian *monocotyledonae*. Micronesica.

[CR82] Fournet J (2002) Flore illustrée des phanérogames de Guadeloupe et de Martinique. Tome 2:1325–2538. La Librairie du Cirad, Montpellier.

[CR83] Foxcroft CL, Henderson L, Nichols GR, Martin BW (2003). A revised list of alien plants for the Kruger National Park. Koedoe.

[CR84] Fraser J (1896) Bicton, Devon. Gardening world January 4:285–286.

[CR85] Fraser-Jenkins CR (2006) The First Botanical Collectors in Nepal - The Fern collections of Hamilton, Gardner and Wallich - lost herbaria, a lost botanist, lost letters, and lost books somewhat rediscovered. Bishen Singh Mahendra Pal Singh, Dehra Dun.

[CR86] Funk N (1859). Plantes Fleuries observées pendent le mois d’Octobre-Serre chaude. Journal D'horticulture Pratique De La Belgique.

[CR87] Galeotti M (ed) (1858) Miscellanées. Journal d’Horticulture Pratique de la Belgique 12:111-112.

[CR88] Gallardo B, Zieritz A, Aldridge DC (2015). The importance of the human footprint in shaping the global distribution of terrestrial, freshwater, and marine invaders. PLoS ONE.

[CR89] GISD Global Invasive Species Database (2021) 100 of the World's Worst Invasive Alien Species. *Hedychium gardnerianum.* http://www.iucngisd.org/gisd/100_worst.php. Accessed 21 Jun 2021.

[CR90] González-Lazo E (2011) Manifestación de Impacto Ambiental Modalidad Particular - Sector hidráulico: Canal De Riego Rio Bravo, Construcción de un Canal de Tierra para conducir Aguas del Rio Caribe, para Riego de Cultivos de Arroz en Ejido El Tigre. Municipio de Candelaria, Estado de Campeche. https://docplayer.es/81387936-Manifestacion-de-impacto-ambiental-modalidad-particular-sector-hidraulico.html.

[CR146] González-Torres LR, Rankin R, Palmarola A (Eds.) (2012) Plantas invasoras en Cuba. Bissea 6(NE1): 1–138. http://repositorio.geotech.cu/jspui/handle/1234/520.

[CR91] Goodland T, Healey JR (1996). The invasion of Jamaican montane rainforests by the Australian tree *Pittosporum undulatum*.

[CR92] Government of Japan (2012) List of candidate species of plants (that are considered to be candidates from the existing literature) to the list of invasive alien species. https://www.env.go.jp/nature/intro/2outline/koudou/gyoukai/ref1-1.pdf. Accessed 21 Jun 2021.

[CR93] Government of Trinidad and Tobago (2021) List of Trinidad and Tobago Invasive Alien Species (IAS). http://www.biodiversity.gov.tt/index.php/trinidad-a-tobago-biodiversity/invasive-alien-species.html. Accessed 21 Jun 2021.

[CR94] Graham R (1825). List of rare plants which have flowered in the Royal Botanic Garden, Edinburgh, during the last three months. Edinburgh Philos J.

[CR95] Greenwood E, Lyus S, Lampert R (2018). Liverpool botanic garden: early curators and gardeners. Trans Hist Soci Lancas Ches.

[CR96] Grisebach AHR (1864). Flora of the British West Indian Islands.

[CR97] Grubb PJ, Tanner EVJ (1976). The montane and soils of Jamaica: A reassessment. Journal of the Arnold Arboretum.

[CR98] Gubhaju MR, Gaha Y (2019). Ethnomedicinal uses of plants in Mityal, Palpa, Nepal. J Pl Res.

[CR99] Guilfoyle WR (1883). Catalogue of plants under cultivation in the Melbourne Botanic Gardens, alphabetically arranged.

[CR100] Haddawaya NR, Baylissb HR (2015). Shades of grey: two forms of grey literature important for reviews in conservation. Biol Cons.

[CR101] Gardeners' Chronicle (ed) (1892) *Hedychium gardnerianum* -Supplementary Sheet. The Gardeners' chronicle: a weekly illustrated journal of horticulture and allied subjects February 6: Photograh 178.

[CR102] Gardeners' Chronicle (ed) (1875) *Hedychium gardnerianum*. The Gardeners' chronicle: a weekly illustrated journal of horticulture and allied subjects April 10:461.

[CR103] Henderson L (2001) A complete guide to declared weeds and invaders in South Africa, including another 36 species invasive in that region. Plant Protection Research Institute Handbook No. 12 Alien Weeds and Invasive Plants. Agricultural Research Council, Paarl Printers, Cape Town.

[CR104] Henriques JA (1879). Catalogo das plantas cultivadas no Jardim Botânico da Universidade de Coimbra no anno de 1878.

[CR105] Hequet V, Le Corre M, Rigault F, Blanfort V (2009) Les espèces exotiques envahissantes de Nouvelle-Calédonie. IRD, IAC, Province Nord et Province Sud de Nouvelle-Calédonie.

[CR106] Herrera K, Lorence D, Flynn T, Balick M (2010) Checklist of the Vascular Plants of Pohnpei, Federated States of Micronesia with Local Names and Uses. Allertonia 10:1–192. http://www.jstor.org/stable/23193787.

[CR107] Hill W (1875). Catalogue of the plants in the Queensland Botanic Gardens.

[CR108] Ho PH (1993). An illustrated flora of Vietnam.

[CR109] Hogg R (1880). Hedychium gardnerianum. J Hortic Cottag Garden Home Farm.

[CR110] Hooker WJ (1825). A catalogue of plants contained in the Royal Botanical Garden of Glasgow in the year 1825 alphabetically arranged.

[CR111] Hooker JD (1853). The botany of the Antarctic voyage of HM discovery ships Erebus and Terror in the Years 1839–1843; II Flora Novae-Zelandiae Pat I, Flowering Plants. Lords Commissioners of the Admiralty.

[CR112] Hooker JD (1887) *Hedychium gardnerianum*. Curtis’s Botanical Magazine, Tab. 6913.

[CR113] Horaninow P (1862). Prodromus Monographiae Scitaminearum Additis Nonnullis de Phytographia, de Monocotyleis et Orchideis.

[CR114] Hovey and Co. Horticulturists (1854) Catalogue of green-house and hardy plants, containing choice collections of azaleas, camellias, chrysanthemums, gloxinias, fucshias, cinerarias, verbenas, & c., and carnations, daisies, picotees, phloxes, paeonies, pansies. Press of Hallworth, Boston.

[CR115] Hovey CM (1834) Report of the Committee appointed to name and label the plants and flowers exhibited at Fanueil Hall on the 17th, 18th, and 19th September. In: Gray JC (ed) An Address Delivered Before the Massachusetts Horticultural Society, at their Sixth Anniversary. Printed by J.T. Buckingham, Boston, pp 22–26.

[CR116] Hoveys and Co (1871). Hoveys' illustrated catalogue of new plants for 1871.

[CR117] Hoveys and Co. (1872) Hoveys' illustrated catalogue of new plants for 1872. Boston.

[CR118] Howell C (2008) Consolidated list of environmental weeds in New Zealand**.** Department of Conservation. DOC Research & Development Series 292. Science & Technical Publishing, Wellington.

[CR119] Hulme PE (2011). Addressing the threat to biodiversity from botanic gardens. Trends Ecol Evol.

[CR120] Hyde MA, Wursten BT, Ballings P, Coates PM (2021) Flora of Zimbabwe. https://www.zimbabweflora.co.zw/speciesdata/species.php?species_id=116120. Accessed 21 Jun 2021.

[CR121] I3N Brasil (2021) Base de dados de espécies exóticas invasoras do Brasil, Instituto Hórus de Desenvolvimento e Conservação Ambiental, Florianópolis–SC. http://bd.institutohorus.org.br/www. Accessed 21 Jun 2021.

[CR122] IHU Institute des Herbiers Universitaires (2021). *Hedychium gardnerianum*. https://science.mnhn.fr/institution/clf/collection/clf/item/clf082528. Accessed 21 Jun 2021.

[CR123] Internet Archive (2021) Digital Library of Free & Borrowable Books. https://archive.org/. Accessed 21 Jun 2021.

[CR124] India Biodiversity Portal (2021). *Hedychium gardnerianum*. https://indiabiodiversity.org/species/show/229894. Accessed 21 Jun 2021.

[CR125] Integrated Taxonomic Information System (2021) ITIS Search. https://www.itis.gov/servlet/SingleRpt/SingleRpt. Accessed 21 Jun 2021.

[CR126] IPNI (2021) International Plant Names Index. https://www.ipni.org/n/60474165-2. Accessed 21 Jun 2021.

[CR127] Iremonger S (2002). A Guide to Plants in the Blue Mountains of Jamaica.

[CR129] Jain SK, Prakash V (1995). *Zingiberaceae* of India: phytogeography and endemism. Rheedea.

[CR130] Junior O (1872). *Hedychium gardnerianum* Wall. Jornal De Horticultura Prática.

[CR131] Kaye T (1820) The stranger in Liverpool; or an historical and descriptive view of the town of Liverpool and its environs. 6th edition. T. Kaye, Liverpool.

[CR132] Ker-Gawler JB (1824) *Hedychium gardnerianum* Mr. Gardner's garland-flower. Bot. Reg. 9, plate 774, 774A and 774B.

[CR133] Kew Royal Botanic Gardens (2021) The Herbarium Catalogue. http://www.kew.org/herbcat. Accessed 21 Jun 2021.

[CR134] Khan MS, Rahman MM, Ali MA (2001). Red Data Book of Vascular Plants of Bangladesh.

[CR135] Kuester LB, Regel E, Rach L, Herder F (1858) Index Seminum, quae Hortus Botanicus Imperialis Petropolitanus pro mutua commutatione offert, Accedunt animadversiones botanicae nonnullae. Petropoli.

[CR136] Kull CA, Alpers E, Tassin J (2015). Marooned plants: vernacular naming practices in the Mascarene Islands. Environ Hist.

[CR137] Lambdon P, Darlow A (2008) Botanical Survey of Ascension Island and St. Helena. South Atlantic Invasive Species Project. RSPB, EC.

[CR138] Law S (2007). Liverpool/Calcutta Exchanges: William Roscoe's Reappraisal of the First Linnaean Order of Plants. Gard Hist.

[CR139] Lawrence A, Thomas J, Houghton J, Weldon P (2015). Collecting the evidence: improving access to grey literature and data for public policy and practice. Aust Acad Res Libr.

[CR140] Lehmann JGC (1834) Delectus seminum quae in horto Hamburgensium botanico. Typis Ioannis Augusti Meissneri. Hamburgi.

[CR141] Levy-Yamamori R, Taaffe G (2004). Garden Plants of Japan.

[CR142] Lindley J, Pursh F, Donn J (1823) Hortus Cantabrigiensis Cambridge Botanic Garden. 10th Edition. London: Richard Taylor Printer.

[CR143] Lindley J, Pursh F, Donn J (1826) Hortus Cantabrigiensis. Cambridge Botanic Garden (ed). 11th Edition. Richard Taylor Printer, London.

[CR144] Galeotti M (ed) (1853–1854) Société Royale Linnéenne de Bruxelles, Neuvième Exposition Publique. Journal d’Horticulture Pratique de la Belgique 11:250–253.

[CR145] Lowe RT (1857) A manual flora of Madeira and the adjacent islands of Porto Santo and the Desertas. John Van Voorst, London. Part I: i-xii, 1–106.

[CR147] Ludlam A (1865). Essay on the cultivation and acclimatization of trees and plants. Trans Proc Royal Soc N Z.

[CR148] Maas PJM, Maas H (1990). Flora Vascular de la Isla Española: Zingiberaceae. Moscosoa.

[CR149] Macdonald IAW (1991) Conservation implications of the invasion of Southern Africa by alien organisms. PhD Thesis, University of Cape Town.

[CR150] MacKee HS (1994). Catalogue des plantes introduites et cultivées en Nouvelle-Calédonie.

[CR151] Marçon SL (2009). Composição florística e estrutura do componente arbustivo-arbóreo do Parque Natural Municipal da Cratera de Colônia, São Paulo.

[CR152] Mascarine Cadetiana (2021) Hedychium gardnerianum. https://mascarine.cbnm.org/index.php/flore/index-de-la-flore/nom?code_taxref=639091. Accessed 21 Jun 2021.

[CR153] Martius CFP, Eichler AG (Eds.) (1890) Flora Brasiliensis, enumeratio plantarum in Brasilia hactenus detectarum. Vol 3, Pars 3. Frid. Fleischer in Comm, Leipzig.

[CR154] Matuda E (1950). A contribution to our knowledge of wild flora of Mt. Ovando. Am Midland Nat.

[CR155] McCormack G (2013) Cook Islands Biodiversity Database, Version 2007.2. Cook Islands Natural Heritage Trust, Rarotonga. http://cookislands.bishopmuseum.org. Accessed 21 Jun 2021.

[CR156] Medeiros ABC (1865) Catálogo das plantas de A. B. da C. M. Manuscript list at Ponta Delgada. Public Library and Regional Archive

[CR157] Medeiros JR, Campos LB, Mendonça SC, Davin LB, Lewis NG (2003). Composition and antimicrobial activity of the essential oils from invasive species of the Azores *Hedychium gardnerianum* and *Pittosporum undulatum*. Phytochemistry.

[CR158] Menéndez F (1923). Catálogo de las plantas existentes en el jardín de aclimatación de La Orotava (Canarias) Ministerio de Fomento, Dirección General de Agricultura y Montes.

[CR159] Menezes CA (1894) Catálogo das Phanerogâmicas da Madeira e do Porto Santo não indicadas na Flora destas Ilhas, do Revdº Padre Richard Thomas Lowe. Funchal.

[CR160] Menezes CA (1914) Flora do Arquipelago da Madeira (Phanerogamicas e Cryptogamicas vasculares). Junta Agricola da Madeira, Typ. Bazar do Povo, Funchal.

[CR161] Messrs V and Sons (1870) Choice Seed. Subtropical and ornamental foliage plants, Royal Exotic Nursery, Chelsea, S.W. The Gardners’ Chronicle and Agricultural Gazette February 19:238.

[CR162] Meyer FS, Schwirkowski P (2019). Checklist de angiospermas da APA Municipal do Rio Vermelho/Humboldt, Santa Cat arina, Brasil. Rodriguésia.

[CR163] Minden V, Hennenberg KJ, Porembsk IS, Boehmer HJ (2010). Invasion and management of alien *Hedychium gardnerianum* (kahili ginger, *Zingiberaceae*) alter plant species composition of a montane rainforest on the island of Hawai’i. Plant Ecol.

[CR164] Minden V, Jacobi JD, Porembski S, Boehmer HJ (2010). Effects of invasive alien kahili ginger (*Hedychium gardnerianum*) on native plant species regeneration in a Hawaiian rainforest. Appl Veg Sci.

[CR165] MNHN Muséum national d’Histoire naturelle (2021) Specimen P06136196. https://science.mnhn.fr/institution/mnhn/search. Accessed 21 Jun 2021

[CR166] Molina A (1975). Enumeración de las plantas de Honduras. Ceiba.

[CR167] Napper W (1892) Tregullow. The Gardeners' chronicle: a weekly illustrated journal of horticulture and allied subjects March 12:342.

[CR168] Natural History Museum (2021) Data Portal: Specimen BM000574691 https://data.nhm.ac.uk/object/d8a98ec0-f4fa-4188-999a-0e4fba93bccc/1600300800000. Accessed 21 Jun 2021.

[CR169] Nel JL, Richardson DM, Rouget M, Mgidi TN, Mdzeke N, Le Maitre DC, van Wilgen BW, Schonegevel L, Henderson L, Neser S (2004). A proposed classification of invasive alien plant species in South Africa: towards prioritizing species and areas for management action. S Afr J Sci..

[CR170] Nepal National Herbarium and Plant Laboratories KATH (2021) https://plantdatabase.kath.gov.np/plants/hedychium-gardnerianum-sheppard-ez-ker-gawl-KATH018274. Accessed 13 Oct 2020.

[CR171] New Zealand Government (2020) National Pest Plant Accord Manual. New Zealand.

[CR172] Nirola S, Das AP (2017). Endemic monocot flora of Darjeeling Himalaya, West Bengal, India. Pleione.

[CR173] Noltie HJ (1994). Flora of Bhutan including Record of plants from Sikkim and Darjeeling.

[CR174] Nunes H, Falé PL, Duarte MF, Serralheiro ML, Borba AES, Silva JFM (2014). *Pittosporum undulatum* and *Hedychium gardnerianum*: nutritive value and secondary metabolites on cattle reproductive performances. Int J Pure Appl Sci Technol.

[CR175] Pagad S, Wong LJ (2020) Global Register of Introduced and Invasive Species – Ascension Island, Saint Helena, Ascension and Tristan da Cunha. Version 1.2. Invasive Species Specialist Group ISSG. Checklist dataset. doi:10.15468/vijxee.

[CR176] PAHSMA Port Arthur Historic Site Management Authority (2014) Government Gardens Plant guide. https://portarthur.org.au/wp-content/uploads/2019/05/Government_Garden_Guide.pdf. Accessed 21 Jun 2021.

[CR177] Pfitzer W (1866) Preis verzeichnis über samen und pflanzen. Stuttgart

[CR178] Philippi F (1884) Memoria i catálogo de las plantas cultivadas en el Jardín Botánico hasta el 1º. de Mayo de 1884. Jardín Botánico de Santiago (ed). Imprenta Nacional, Moneda, 112. Santiago de Chile.

[CR179] PIER Pacific Island Ecosystems at Risk (2021) *Hedychium gardnerianum*. http://www.hear.org/pier/species/hedychium_gardnerianum.htm. Accessed 21 Jun 2021.

[CR180] POWO Plants of the World Online (2021). *Hedychium gardnerianum*. http://plantsoftheworldonline.org/taxon/urn:lsid:ipni.org:names:69077-3. Accessed 21 Jun 2021.

[CR181] Prestoe H (1869) Catalog of Plants Cultivated in the Royal Botanic Gardens. Proceedings of the Scientific Association of Trinidad Part VI:251–355.

[CR182] Ramil-Rego P, Vales C (ed) (2019) Especies Exóticas Invasoras: situación e propuestas de mitigación. Monografías do IBADER - Serie Biodiversidade.

[CR183] Rare Exotic Seeds (2021) 100 *Hedychium gardnerianum* seeds (Kahili Ginger Seeds) USD 14.00. https://www.rarexoticseeds.com/en/hedychium-gardnerianum-seeds-kahili-ginger-seeds.html. Accessed 21 Jun 2021.

[CR184] Reflora (2021) Herbário Virtual. http://reflora.jbrj.gov.br/reflora/herbarioVirtual/. Accessed 21 Jun 2021.

[CR185] Regel E, Herder F (1860–61/63–66) Index Seminum, quae Hortus Botanicus Imperialis Petropolitanus pro mutua commutatione offert, Accedunt animadversiones botanicae nonnullae. Petropoli.

[CR186] Borbonica - Réunion (2021) Portail SINP de la Réunion. https://www.borbonica.re/. Accessed 21 Jun 2021.

[CR187] Reyes-Betancort JA, Pérez-de-Paz PL (2001) Contribución al estúdio de la flórula del Parque García Sanabria (Santa Cruz de Tenerife, Tenerife. Islas Canarias). Rve. Acad. Canar. Cienc. XII(3–4):169–190.

[CR188] Richard JMC (1856). Catalogue des Végétaux Cultivés au Jardin du Gouvernement a l'Ile de la Réunion.

[CR189] Rodriguez JA (1990). Flores de Canarias.

[CR190] Rosa JS, Mascarenhas C, Oliveira L, Teixeira T, Barreto MC (2010). Biological activity of essential oils from seven Azorean plants against *Pseudaletia unipuncta* (Lepidoptera: Noctuidae). J Appl Entomol.

[CR191] Roscoe W (1828) Monandrian plants of the order Scitamineae: chiefly drawn from living specimens in the botanic garden at Liverpool, arranged according to the system of Linnaeus with descriptions and observations. Printed by George Smith, Liverpool.

[CR192] Roscoe H (1833) The life of William Roscoe, by his son Henry Roscoe. Boston, Russell, Odiorne, and Company. Two Volumes.

[CR193] Rösner K (1900). Beitrag zur Bepflanzung dekorativer Gruppen in Gartenanlagen. Die Gartenwelt.

[CR194] Royal Botanic Garden Edinburg (2021) https://data.rbge.org.uk/search/herbarium/. Accessed 21 Jun 2021.

[CR195] Royal Horticultural Society (2021) https://www.rhs.org.uk/Plants/8427/i-Hedychium-gardnerianum-i/Details. Accessed 21 Jun 2021.

[CR196] Sanchez-Ruiz A (2005). Cuba: Parque Nacional La Bayamesa. Rapid Biological Inventories.

[CR197] Sanoj E (2011) Taxonomic revision of the genus *Hedychium* J Koenig *Zingiberaceae* in India. PhD. Thesis. University of Calicut, Kerala. http://hdl.handle.net/10603/80898.

[CR198] Sanoj E, Mamiyil S, Pradeep AK (2013). Circumscription and lectotypification of Hedychium villosum and its variety H villosum var tenuiflorum (Zingiberaceae). PhytoKeys.

[CR199] Seemann B (1852–1857) The botany of the voyage of H.M.S. Herald, under the command of Captain Henry Kellett, R.N., C.B., during the years 1845–51. Lovell Reeve, London.

[CR200] Shagarodsky T, Fuentes V, Barrios O, Castiñeiras L, Fundora Z, Sánchez P, Fernández L, Cristóbal R, García M, Giraudy C (2003). Diversidad de especies alimenticias en tres mercados agrícolas de la Habana, Cuba. Agronomía Mesoamericana.

[CR201] Shepherd J (no date). Manuscript notes. Botanical Garden of Liverpool. World Museum of Liverpool Herbarium. Liverpool.

[CR202] Shrestha KK, Bhattarai S, Bhandari P (2018). Handbook of Flowering Plants of Nepal Gymnosperms and Angiosperms: Cicadaceae-Betulaceae.

[CR203] Silva L, Ojeda-Land E, Rodríguez-Luengo JL (Eds.) (2008) Invasive Terrestrial Flora & Fauna of Macaronesia. TOP 100 in Azores, Madeira and Canaries. ARENA, Ponta Delgada.

[CR204] Silva-Pando FJ, Pino R, Pino JJ, García XR, Morla C, Cebolla C, Gómez F, Camaño JL, Rial S, Álvarez D, Blanco JB, Paz M (2009). Aportaciones a la flora de Galicia, IX. Nova Acta Científica Compostelana (bioloxía).

[CR205] Smith L (1832). Memoir and Correspondence of the Late Sir James Edward Smith, M.D.

[CR206] Smith CW, Stone CP, Scott JM (1985). Impact of alien plants on Hawaii's native biota. Hawai'i's terrestrial ecosystems: preservation and management.

[CR207] Smith RM (1998). Flora of southern Africa contributions 11: *Zingiberaceae*. Bothalia.

[CR208] Smith AC (1979) Flora Vitiensis nova: a new Flora of Fiji (spermatophytes only). Vol. I. Pacific Tropical Botanical Garden, Lawai, Kauai, Hawaii.

[CR209] Soubeyran Y (2008) Espèces exotiques envahissantes dans les collectivités françaises d’outre-mer. Etat des lieux et recommandations. Collection Planète Nature. Comité français de l’UICN, Paris.

[CR210] Southern California Acclimatizing Association (ed) (1897) General Catalogue and Garden Guide for the South. Nº5. Henry G. Gilbert Nursery and Seed Trade Catalog Collection. Santa Barbara, California.

[CR211] Space JC, Flynn T (2002) Report to the Government of the Cook Islands on Invasive Plant Species of Environmental Concern. USDA Forest Service, Honululu, Hawai’i.

[CR212] Stehlé H (1935) Flore de la Guadeloupe et dépendances: Essai d'écologie et de géographie botanique. L'Imprimerie catholique, Basse-Terre - Guadeloupe 1935.

[CR213] Stone CP, Smith CW, Tunison JT (eds.) (1992) Alien plant invasions in native ecosystems of Hawaii: management and research. University of Hawaii, Honolulu, Hawai’i.

[CR214] Strong WC, Muzzey AB, Sturtevant EL (1880) History of the Massachusetts Horticultural Society 1829–1878. Massachusetts Horticultural Society Ed., Boston.

[CR215] Sturtevant ED (1885). Catalogue of Rare Water Lilies and other choice of aquatic plants.

[CR216] Sutherland CHN (2008) Catálogo de las plantas vasculares de Honduras. Espermatofitas: 1–1576. SERNA/Guaymuras, Tegucigalpa, Honduras.

[CR217] Swaziland's Alien Plants Database (2021). *Hedychium gardnerianum*. http://www.sntc.org.sz/alienplants/speciesinfo.asp. Accessed 21 Jun 2021.

[CR218] Sweet R (1826) Sweet's Hortus Britannicus. Part II. James Ridgway, London.

[CR219] Tan DT, Thu PQ, Dell B (2012). Invasive Plant Species in the National Parks of Vietnam. Forests.

[CR220] Tanaka N, Ohi-Toma T, Aung M, Murata J (2016). Systematic notes on the genus *Hedychium* (*Zingiberaceae*) in Myanmar. Bull Natl Mus Nat Sci Ser B..

[CR221] Tangsibji Hydro Energy Limited (2015) BHU: Second Green Power Development Project - 118 MW Nikacchu Hydropower Project. Environmental Safeguard Monitoring Report. https://www.adb.org/sites/default/files/project-document/176339/44444-013-emr-01.pdf. Accessed 21 Jun 2021.

[CR222] Tavares WR, Barreto M, Seca A (2020). Uncharted Source of Medicinal Products: The case of the *Hedychium* genus. Medicines.

[CR223] Tavares-Silva P, Lima LV, Andrade RC, Cabral A, Paula MA, Ferreira FM (2018). Flora vascular exótica e daninha do Jardim Botânico da Universidade Federal de Juiz de Fora, Minas Gerais, Brasil. Pesquisas-Botânica.

[CR224] Tenore M (1845) Catalogo delle piante che si coltivano nell'Orto Botanico Di Napoli. Tipografia dell'Aquila.

[CR128] The Islander (2003) Ascension island - a newcomer's guide. 31st July 2003. http://www.the-islander.org.ac/oldsite/1649.htm. Accessed 21 Jun 2021.

[CR225] The Linnean Society of London (2021) The Linnean Collections. LINN-HS 8.8. *Hedychium gardnerianum* (Herb Smith). University of London. http://linnean-online.org/35722/. Accessed 21 Jun 2021.

[CR226] The Map Archive (2021) British Conquest of India 1753–1914 (Ax01147) https://www.themaparchive.com/product/british-conquest-of-india-17531858/. Accessed 21 Jun 2021.

[CR227] The Plant List (2013) Version 1.1. http://www.theplantlist.org/tpl1.1/record/kew-248143. Accessed 21 Jun 2021.

[CR228] The Wallich Catalogue Online (2021) Number 6550. http://wallich.rbge.info/. Accessed 21 Jun 2021.

[CR229] Tilloch A, Taylor R (1824). Analysis of periodical works on natural history. Philos Mag J.

[CR230] Trelease W (1897) Botanical observations on the Azores. Missouri Botanical Garden. Eighth Annual Report: 77–220. 66 plates.

[CR231] Uddin MZ (2015) Plant diversity assessment in Khadimnagar National Park, Sylhet. Final Report of the Flora. Bangladesh Forest Department, Ministry of Environment and Forest, Bangladesh.

[CR232] UICN|Comité Francais (2017a) Initiative sur les espèces exotiques envahissantes en d’outre-mer. *Hedychium gardnerianum* Martinique. https://especes-envahissantes-outremer.fr/especes_envahissante/hedychium-gardnerianum/. Accessed 21 Jun 2021.

[CR233] UICN|Comité Francais (2017b). Initiative sur les espèces exotiques envahissantes en d’outre-mer. *Hedychium gardnerianum* Réunion. https://especes-envahissantes-outremer.fr/especes_envahissante/hedychium-gardnerianum-3/. Accessed 21 Jun 2021.

[CR234] UNE University of New England (2021). Grey literature. https://www.une.edu.au/library/support/eskills-plus/research-skills/grey-literature. Accessed 21 Jun 2021.

[CR235] USDA Plants DataBase (2021) *Hedychium gardnerianum*. https://plants.sc.egov.usda.gov/core/profile?symbol=HEGA. Accessed 21 Jun 2021.

[CR236] Voigt JO (1845). Hortus suburbanus Calcuttensis; A catalogue of the plants which have been cultivated in the Hon. East India Company's botanical garden, Calcutta, and in the Serampore botanical garden.

[CR237] Walker Art Gallery (2021a) Portrait of William Roscoe (1753–1831), artist John Williamson. Image use: CC BY-NC. https://artuk.org/discover/artworks/william-roscoe-17531831-97519. Accessed 21 Jun 2021.

[CR238] Walker Art Gallery (2021b) Portrait of John Shepherd (1764–1836), artist John Williamson. Image use: CC BY-NC. https://artuk.org/discover/artworks/john-shepherd-17641836-97554. Accessed 21 Jun 2021.

[CR239] Walker S (1847) Transactions of the Society. Transactions of the Massachusetts Horticultural Society for the years 1843–4–5–6. Dutton and Wentworth's Print, Boston.

[CR240] Wallich N (1832). Plantae Asiaticae rariores, or Descriptions and figures of a select number of unpublished East Indian plants.

[CR241] Wallich N (1840). Report on the Royal Botanic Garden.

[CR242] Wallich N (1853) Initiatory attempt to define the species of *Hedychium* and settle their synonymy. Hooker’s Journal of Botany and Kew Garden Miscellany, 5: 321–329 and 367–377.

[CR243] Watson W (1896) Foreign correspondence, Mr. Chamberlain Garden. Garden and forest, a journal of horticulture, landscape art and forestry, February 12:63–64.

[CR244] Watson M (2013.08.13) Botanic stories: Wallich Catalogue: Sylhet, Pundua & Khasia Hills. RBG Edinburgh. https://stories.rbge.org.uk/archives/5029. 21 Jun 2021.

[CR245] Welcome Collection (2021) Portrait of Nathaniel Wallich (1786–1854) in 1833, artist John Lucas. Image use: CC BY 4.0. https://wellcomecollection.org/works/a8bhp2dr/images?id=ng832p8v. Accessed 21 Jun 2021.

[CR246] Wilks W, Weathers J (1896). Appendix II A selected list of the native and Introduced plants observed in the Botanic Garden near Orotava, Tenerife, in March, 1893. J Roy Hort Soc.

[CR247] Williams PA, Winks C, Rijkse W (2003). Forest processes in the presence of wild ginger (*Hedychium gardnerianum)*. New Zeal J Ecol.

[CR248] Witt A, Luke Q (2017) Guide to the naturalized and invasive plants of Eastern Africa. [ed. by Witt A, Luke Q]. Wallingford. http://www.cabi.org/cabebooks/ebook/20173158959. Accessed 21 Jun 2021.

[CR249] Wm R Prince & Co (1830) Periodical catalogue of greenhouse shrubs, vines, herbaceous plants, and bulbous roots cultivated and for sale at the Linnaean Botanic Garden. New York.

[CR250] Wongsuwan P, Picheansoonthon C (2011). Taxonomic revision of the genus *Hedychium* J. Koenig *(Zingiberacea*e) in Thailand (part 1). J Royal Inst Thail.

[CR251] Wongsuwan P, Picheansoonthon C (2012). Taxonomic revision of the genus *Hedychium* J. Koenig *(Zingiberacea*e) in Thailand (part 2). J Royal Inst Thail.

[CR252] Wood JM (1895) Report on Natal Botanic Gardens and Colonial Herbarium for the year 1894. Durban Botanic Society (ed), Natal Mercury Steam Printing Works.

[CR253] Wood TH, Whitten WM, Williams NH (2000). Phylogeny of *Hedychium* and related genera (*Zingiberaceae*) based on its sequence data. Edinb J Bot.

[CR254] Wu ZY, Raven PH (eds.) (2000) Flora of China. Vol. 24 (*Flagellariaceae* through *Marantaceae*). Science Press, Beijing, and Missouri Botanical Garden Press, St. Louis.

